# Bullshit-sensitivity predicts prosocial behavior

**DOI:** 10.1371/journal.pone.0201474

**Published:** 2018-07-31

**Authors:** Arvid Erlandsson, Artur Nilsson, Gustav Tinghög, Daniel Västfjäll

**Affiliations:** 1 Linköping University, Department of Behavioural Sciences and Learning, Linköping, Sweden; 2 Lund University, Department of Psychology, Lund, Sweden; 3 Linköping University, Department of Management and Engineering, Linköping, Sweden; 4 Decision Research, Eugene, OR, United States of America; University of California Los Angeles, UNITED STATES

## Abstract

Bullshit-sensitivity is the ability to distinguish pseudo-profound bullshit sentences (e.g. “Your movement transforms universal observations”) from genuinely profound sentences (e.g. “The person who never made a mistake never tried something new”). Although bullshit-sensitivity has been linked to other individual difference measures, it has not yet been shown to predict any actual behavior. We therefore conducted a survey study with over a thousand participants from a general sample of the Swedish population and assessed participants’ bullshit-receptivity (i.e. their perceived meaningfulness of seven bullshit sentences) and profoundness-receptivity (i.e. their perceived meaningfulness of seven genuinely profound sentences), and used these variables to predict two types of prosocial behavior (self-reported donations and a decision to volunteer for charity). Despite bullshit-receptivity and profoundness-receptivity being positively correlated with each other, logistic regression analyses showed that profoundness-receptivity had a positive association whereas bullshit-receptivity had a negative association with both types of prosocial behavior. These relations held up for the most part when controlling for potentially intermediating factors such as cognitive ability, time spent completing the survey, sex, age, level of education, and religiosity. The results suggest that people who are better at distinguishing the pseudo-profound from the actually profound are more prosocial.

## Introduction

Seemingly impressive statements that are presented as meaningful or true but are actually vacuous (e.g. “Good health imparts reality to subtle creativity”) have been called *pseudo-profound bullshit* [1]. Bullshit-statements are (a) constructed absent any concern for the truth and (b) do not consistently have any unambiguous meaning [1, 2]. Although bullshit has existed for centuries, academic interest in the philosophical analysis of bullshit [3–5], and empirical studies of bullshit [1, 6] are new phenomena.

Recently, some psychological research has focused on individual differences in the extent to which people perceive bullshit as meaningful. These studies have shown that people who rate bullshit sentences as highly meaningful have more religious and supernatural beliefs, are less reflective, intelligent, and numerate, more prone to ontological confusions and conspiratorial ideation [1], endorse free market policies more [7, 8], and have more favorable views of Republican presidential candidates in US politics [6]. The aim of this study is to develop the academic field of bullshit further.

### Bullshit-receptivity, profoundness-receptivity and bullshit-sensitivity

As noted by Pennycook and colleagues [1], the tendency to perceive bullshit sentences as meaningful, which is called *bullshit-receptivity*, is different from *bullshit-sensitivity*, which refers to the ability to distinguish bullshit sentences from genuinely profound sentences (e.g. “A river cuts through a rock, not because of its power but its persistence”). Perceived meaningfulness of genuinely profound sentences and bullshit sentences are generally positively related (e.g. *r* = .38 and *r* = .43 in [1]; and *r* = .52 in [6]), which means that bullshit-receptivity could reflect either a general inclination to perceive *any* sentence or statement as meaningful *or* a propensity to perceive specifically bullshit sentences as meaningful. Bullshit-sensitivity, on the other hand, represents the ability to tell apart the pseudo-profound from the actually profound. In Pennycook’s original article [1], two of the four studies included bullshit-sensitivity calculated by subtracting bullshit-receptivity from *profoundness-receptivity* (i.e. the perceived meaningfulness of genuinely profound sentences), and although it was associated with lower paranormal belief, the results regarding bullshit-sensitivity were less conclusive than those regarding bullshit-receptivity. Because there are inherent scaling problems with variables made out of difference-scores [9], we opted to focus on bullshit-receptivity and profoundness-receptivity in the analyses, inferring bullshit-sensitivity indirectly. If both bullshit- and profoundness-receptivity are positively (or negatively) associated with e.g. prosocial behavior, this suggests that it is the general inclination to perceive anything as meaningful that relates to prosocial behavior. On the contrary, if bullshit-receptivity and profoundness-receptivity predict prosocial behavior in opposite directions, this suggests that it is the ability to distinguish bullshit from the actually profound (i.e. bullshit-sensitivity) that relates to prosocial behavior.

### Reactions to bullshit, prosocial behavior and reflective thinking

Neither bullshit-receptivity nor bullshit-sensitivity have been empirically linked to any behavioral outcome yet. While introducing reliable scales for measuring perceptions of bullshit is an important first step, the value of such scales would increase considerably if they could be linked to important behavioral outcomes. Therefore, the current study investigates whether bullshit-receptivity, profoundness-receptivity (and indirectly bullshit-sensitivity) predict prosocial behavior. We chose prosocial behavior because it represents a characteristically moral, and frequently studied behavior that is perceived as normatively good from all of the main philosophical perspectives of morality. In order to increase generalizability, we operationalized prosocial behavior in two different ways: *Donation experience*, which represents whether the person had donated to charity anytime over the past year, and *Volunteering decision*, which represents whether the person agreed to “volunteer for charity” when completing the survey.

Due to the lack of research on the psychology of bullshit, it is admittedly difficult to predict how bullshit-sensitivity will relate to prosocial behavior. However, higher bullshit-sensitivity (and lower bullshit-receptivity) has proved to be associated with a willingness to engage in analytical and reflective thinking ([1, 8]), and some past research has indeed investigated the relation between reflective thinking and prosocial behavior.

On the one hand, the traditional view of *Homo Economicus* rests on the idea that decisions are based on deliberative reasoning where benefits and costs are weighed against each other and individuals choose the course of action that brings the most favorable consequences for themselves. By extension, more reflective people should be better at detecting bullshit as well as less likely to help others as long as there are no social or economic benefits for themselves. In line with this, Kanazawa and Fontaine [10] found that persons with higher cognitive ability engaged in more free-riding in a one-shot prisoner’s dilemma and Nielsen et al. [11] observed that those with high scores on the cognitive reflection test (CRT; [12]) were more likely to be free-riders in a large-scale public goods game. Also, according to the social-heuristic hypothesis [13, 14], reflective thinking should lead to more selfishness. The explanation for this is that our evolved intuitions are cooperative but that people high on reflective thinking can evaluate more complex trade-offs between selfish and unselfish concerns.

On the other hand, Moore and Loewenstein [15] argue that selfishness is automatic and often unconscious, whereas recognizing and adhering to one’s moral and professional obligations involves more reflective thinking. There are several studies showing that cognitive abilities such as intelligence are positively related to prosocial preferences [16–18], and reflective ability as measured with the CRT specifically has been linked to more “mild altruism” (helping at low personal cost) but less “strong altruism” (helping at high personal cost; [19, 20]). CRT-scores have also been found to predict trust [21], and one study found that people scoring high on the CRT cooperated more in a one shot public goods game, but that this effect disappeared when participants thought that they played against a computer or in the presence of time-pressure [22]. Moreover, several large-scale studies have not been able to detect any link between time pressure and prosocial behavior [23–25], thus casting doubt on the social-heuristic hypothesis.

In sum, because previous theory and research does not give us strong reasons for predicting a direct relation between reactions to bullshit and prosocial behavior, our investigation of this link is admittedly exploratory. There are however, theoretical arguments and empirical studies indicating both a negative and a positive relation between reflective thinking and prosocial behavior, and past research clearly suggests that individual differences in reactions to bullshit are related to reflective thinking [1, 8], which in turn suggests that bullshit-receptivity and/or bullshit-sensitivity could be related to prosocial behavior indirectly. We therefore included reflective thinking as a control variable when empirically investigating the relation between bullshit- and profoundness-receptivity and prosocial behavior.

### Controlling for other Variables

A significant bivariate correlation between how people perceive bullshit and their prosocial behavior would, in itself, be a novel and interesting finding. However, as seen above, bullshit-receptivity, bullshit-sensitivity and prosocial behavior are all known to correlate with other individual difference constructs [1, 6, 7] so it is advisable to take these constructs into consideration as well, and investigate whether bullshit-receptivity or sensitivity has an association with prosocial behavior that cannot be accounted for by control variables. For this reason we measured demographics, religiosity and political orientation on a left-right scale, as well as cognitive ability (numeracy and propensity to engage in reflective thinking as measured with the CRT). We also measured the total amount of time it took for participants to fill out the survey, which served as a rough proxy for the amount of effort they put in when completing the survey.

### The cultural context

Research on reactions to bullshit has to date exclusively used samples of American undergraduate students or Mechanical Turk users, which often are WEIRD (i.e. exclusively from western, educated, industrialized, rich and democratic societies) and therefore far from representative for humanity in general [26]. Although the current study cannot address this problem completely, we did make an effort to recruit participants from a heterogeneous non-American sample, including participants recruited from a roughly nationally representative Swedish online panel.

## Method

### Participants and material

The data were collected as part of a broader survey in collaboration with CMA Research, which is an independent research firm. In order to obtain high statistical power, we requested complete responses from 1000 participants (a post hoc estimate of statistical power using G*Power indicated that 1000 participants would give us 99.8% power to detect a correlation of 0.15. The final sample consisted of 506 women and 509 men (*M*_age_ = 48.94, *SD* = 15.11). The survey included 11 sections that were completed in a fixed order. Several sections in the survey were included as part of other research-projects that are not directly linked to the current research question and therefore reported elsewhere [27–29]. To maximize transparency, the online supplementary information include a complete description of the full survey and the raw data with all variables included in the full survey.

The Swedish law concerning the Ethical Review of Research Involving Humans (SFS 2003:460) serves to protect individuals and human dignity when research is conducted. In accordance with this act and based on the information on the Swedish Ethical Committee homepage, it was concluded that a formal assessment was not necessary because the participants were given full-disclosure of the procedure (i.e., there was no deceit), participants received a payment proportionate to the task, the experimental procedure was noninvasive, and the results were analyzed on a group-level and no responses could be linked to any specific person. Furthermore, all participants were above the age of 18, had agreed to be contacted by CMA research, and voluntarily signed up for participation in this specific survey. They were informed that participation was voluntary and anonymous and that they could withdraw from the experiment at any time. In order to maintain participants’ anonymity and personal integrity, we did not obtain written consent.

#### Bullshit-receptivity and profoundness-receptivity (collected in Section 5)

Participants were asked to read 14 sentences that were inspired by the ones used by Pennycook et al. [1] and respond to how “meaningful and worth considering” each sentence was. They did so on a Likert scale ranging from 1 = *not at all meaningful* to 6 = *very meaningful*. Seven of the sentences represented bullshit. They were presented in a mixed order along with seven genuinely profound sentences (see Table 1 for all sentences). Perceived meaningfulness of the aggregated bullshit sentences (i.e. bullshit-receptivity, *α* = .82) and perceived meaningfulness of the aggregated genuinely profound sentences (i.e. profoundness-receptivity, *α* = .89) correlated positively at *r* = .21, which is significant (*p* < .001) but slightly weaker than comparable estimates from past studies [1, 6]. For the bivariate correlations, we also calculated participants’ bullshit-sensitivity by subtracting their bullshit-receptivity from their profoundness-receptivity (as in [1]).

**Table 1:** Mean perceived meaningfulness of the bullshit-sentences and genuinely meaningful sentences (translated from Swedish).

	Mean (SD) Scale 1–6
**Bullshit sentences (*α* = .82)**	
The hidden meaning transforms the abstract beauty. [2]	2.62 (1.26)
The future elucidates irrational facts for the seeking person. [3]	2.71 (1.29)
Health and tolerance provides creativity for the future. [5]	3.04 (1.42)
Your movement transforms universal observations. [8]	2.42 (1.32)
The whole silence infinite phenomena. [10]	2.52 (1.35)
The invisible is beyond all new immutability. [12]	2.48 (1.37)
The unexplainable touches on the inherent experiences of the universe [13]	2.52 (1.40)
**Genuinely profound sentences (*α* = .89)**	
A river cuts through a rock, not because of its power but its persistence. [1]	4.12 (1.38)
You are not only responsible for the things you say, but also for the things you do not say. [4]	4.37 (1.36)
We have others flaws before our eyes, but our own flaws behind our back. [6]	4.20 (1.35)
Your teacher can open the door, but you have to step in. [7]	4.55 (1.30)
The person who never made a mistake never tried something new. [9]	4.53 (1.29)
Imagined pain does not hurt less because it is imagined [11]	3.74 (1.36)
It is one thing to be tempted but quite another to fall for the temptation [14]	4.30 (1.27)

*Note*. Numbers in brackets show the presentation order of the sentences.

#### Prosocial behavior (Section 11 & 9)

The main dependent variable was measured in two ways. *Donation experience* was measured in Section 11 with the item “Have you donated money to any charity organization during the past year”. Participants could answer *NO* = 0 or *YES* = 1 (71% responded YES).

*Volunteering decision* was measured in Section 9. Participants were told that there were only a few questions left and that they could choose either to “*Go to the final questions*” = 0 or “*Continue for charity*” = 1. They were told that continuing for charity meant that they then would respond to additional survey questions and that 5 SEK (≈ $0.6) would be donated to a charity organization of their choosing. Participants who chose to continue for charity (58.4%) responded to additional questions and could then pick which charity organization they wished to donate to from a list of well-known Swedish organizations. For each participant continuing for charity, we later donated 5 SEK to the charity of their choice.

#### Demographics (Section 3 & 11)

Participants’ *sex* (*Female* = 0, *Male* = 1), current *age*, level of highest completed *education* (1 = *not completed elementary school*; 5 = *university/college degree*), and frequency of religious activities (1 = *never*; 8 = *every day*) were measured in the beginning of Section 11. The religious activity item was highly correlated with three items about religious identity and religious beliefs that were included in Section 3. These four items were therefore aggregated to make up a composite index of *religiosity* (*α* = .80).

#### Political left-right self-placement (Section 11)

Participants were asked to position themselves politically on a scale ranging from 1 = *very far to the left* to 9 = *very far to the right*.

#### Numeracy and cognitive reflection (Section 4)

Participants responded to three numeracy questions taken from Schwartz et al. [30] and from the Berlin numeracy test ([31]), and immediately thereafter to the three questions that make up the original CRT [12]. On both these variables, participants obtained a score between 0 and 3 representing the number of correct responses.

#### Time spent

We also calculated each participant’s total amount of time spent completing the entire survey. This variable was included as a control because participants likely differed in how much effort they put into completing the survey. It should be noted however, that an affirmative answer on the volunteering decision unavoidably prolonged the survey, so a positive correlation between the volunteering decision and time spent completing the survey could be a methodological artefact. In order to reduce the influence of outliers, time scores for participants who used more than an hour to complete the survey were adjusted to 60 minutes but we put no lower limit on time spent. On average, participants spent 30.76 minutes (*SD* = 16.01) completing the survey.

## Results

### Bivariate correlations

The bivariate rank-order correlations between all included variables can be seen in Table 2. Age, level of education, numeracy, reflective ability and time spent completing the survey all correlated positively with profoundness-receptivity, but negatively with bullshit-receptivity (and as a logical consequence positively with bullshit-sensitivity). Religiosity was positively correlated primarily with bullshit-receptivity. Participants’ sex and their political self-placement were only weakly related to bullshit-receptivity and profoundness-receptivity.

**Table 2:** Bivariate Spearman rank-order correlations of the included variable (N = 1015).

	1.	2.	3.	4.	5.	6.	7.	8.	9.	10.	11.	12	13.
**1.** Bullshit-receptivity	1												
**2.** Profoundness-receptivity	.15***	1											
**3.** Bullshit-sensitivity *(Profoundness-receptivity minus Bullshit-receptivity)*	-.70***	.55***	1										
**4.** Sex (0 = female, 1 = male)	-.05	-.10**	-.03	1									
**5.** Current age	-.11***	.26***	.28***	-.08**	1								
**6.** Education	-.18***	.15***	.25***	-.03	.05	1							
**7.** Religiosity	.34***	.07*	-.24***	-.19***	.01	.01	1						
**8.** Rightwing political self-placement	-.01	.03	.02	.12***	.07*	.01	-.01	1					
**9**. Numeracy (0–3)	-.30***	.16***	.39***	.24***	-.00	.24***	-.23***	.06	1				
**10.** Reflective ability (CRT; 0–3)	-.35***	.16***	.42***	.21***	.01	.26***	-.20***	.04	.64***	1			
**11**. Time spent (0-60min)	-.22***	.28***	.40***	.01	.29***	.18***	.01	.02	.37***	.33***	1		
**12.** Donation experience (0 = NO, 1 = YES)	-.05	.20***	.20***	-.12***	.21***	.15***	.11***	.02	.07*	.11***	.20***	1	
**13.** Volunteering Decision (0 = No, 1 = YES)	-.21***	.23***	.36***	-.09**	.10**	.16***	-.01	-.07*	.24***	.25***	.30***	.18***	1

Note.

*** = *p* < .001

** = *p* < .01

* = *p* < .05

Donation experience and volunteering decision correlated *r*_*s*_ = .18 (*p* < .001), which suggests that the two measures of prosocial behavior are related but far from identical. Both measures of prosocial behavior were positively associated with being female, age, and level of education, as well as with time spent completing the survey. Numeracy and reflective thinking also positively predicted prosocial behavior but primarily when measured with the volunteering decision measure, whereas religiosity predicted only the donation experience measure.

Central to the current study, bullshit-sensitivity was clearly positively associated with both measures of prosocial behavior. People who responded YES to the donation experience question had higher profoundness-receptivity but similar bullshit-receptivity compared to those answering NO. People who responded YES to the volunteering decision question had higher profoundness-receptivity but lower bullshit-receptivity compared to those who answered NO.

### Regression analyses

Models based on logistic regressions were used to test the hypotheses using either donation experience or volunteering decision as the outcome variable (0 = *no helping*; 1 = *helping*). The first model included only bullshit-receptivity and profoundness-receptivity as predictor variables. The second model added cognitive ability (number of correct responses on the numeracy and CRT questions; theoretical range 0–6), whereas the third model added participant’s time spent (in minutes) completing the whole survey. The fourth model added participants’ current age, highest level of completed education and self-rated religiosity, and the fifth and final model added participants’ sex (0 = female, 1 = male) and political self-placement where a higher number indicated a more rightwing political attitude. We explain the results from only Model 1 and 5 in detail below but the results from all models are reported in Tables 3 and 4. We also analyzed the data with Structural Equation Modelling (SEM) to control for the influence of covariates while accounting for measurement error. The results from the SEM (which were very similar to the ones reported here) are presented in S5 File (Appendix A).

**Table 3:** Beta coefficients (standard error), [95% odds ratio estimates] and Cox & Snell Pseudo R^2^ of the models predicting donation experience.

	Model 1	Model 2	Model 3	Model 4	Model 5
Intercept	-0.85 (0.34)*	-0.91 (0.35)*	-1.12 (0.36)**	-2.92 (0.48)***	-3.09 (0.52)***
Profoundness-receptivity (range 1–6)	0.55 (0.08)*** [1.49–2.02]	0.53 (0.08)*** [1.45–2.01]	0.45 (0.09)*** [1.32–1.86]	0.33 (0.09)*** [1.15–1.65]	0.30 (0.09)** [1.13–1.61]
Bullshit-receptivity (range 1–6)	-0.21 (0.07)** [0.70–0.93]	-0.19 (0.08)* [0.71–0.97]	-0.14 (0.80)† [0.75–1.02]	-0.16 (0.09)† [0.72–1.02]	-0.13 (0.09) [0.74–1.04]
Cognitive ability (0–6 correct answers)		0.03 (0.04) [0.95–1.12]	-0.01 (0.04) [0.91–1.08]	0.03 (0.05) [0.94–1.13]	0.06 (0.05) [0.97–1.17]
Time spent (0–60 minutes)			0.02 (0.01)** [1.01–1.03]	0.01 (0.01)* [1.00–1.02]	0.01 (0.01)* [1.00–1.02]
Current age (18–75 years)				0.02 (0.01)*** [1.01–1.03]	0.02 (0.01)*** [1.01–1.03]
Education (1–5)				0.20 (0.07)** [1.06–1.41]	0.18 (0.07)* [1.04–1.39]
Religiosity (1–7)				0.25 (0.06)*** [1.14–1.44]	0.23 (0.06)*** [1.12–1.42]
Sex (0 = female, 1 = male)					-0.42 (0.16)** [0.49–0.90]
Rightwing political attitude (1–9)					0.02 (0.04) [0.94–1.11]
Cox & Snell Pseudo R^2^	5.1%	5.1%	6.2%	10.3%	10.9%

Note

*** = *p* < .001

** = *p* < .01

* = *p* < .05

† = *p* < .10

**Table 4:** Beta coefficients (standard error), [95% odds ratio estimates] and Cox & Snell Pseudo R^2^ of the models predicting volunteering decision.

	Model 1	Model 2	Model 3	Model 4	Model 5
Intercept	-1.14 (0.33)**	-1.50 (0.35)***	-1.80 (0.36)***	-2.32 (0.46)***	-2.01 (0.51)**
Profoundness-receptivity (range 1–6)	0.72 (0.08)*** [1.75–2.40]	0.62 (0.08)*** [1.59–2.19]	0.53 (0.09)*** [1.44–2.01]	0.53 (0.09)*** [1.42–2.02]	0.50 (0.09)*** [1.38–1.97]
Bullshit-receptivity (range 1–6)	-0.58 (0.07*** [0.49–0.64]	-0.45 (0.08)*** [0.55–0.74]	-0.40 (0.08)*** [0.58–0.78]	-0.46 (0.08)*** [0.54–0.75]	-0.43 (0.08) *** [0.55–0.77]
Cognitive ability (0–6 correct answers)		0.18 (0.40)*** [1.11–1.30]	0.14 (0.04)** [1.06–1.25]	0.14 (0.04)** [1.06–1.26]	0.19 [0.05]*** [1.10–1.32]
Time spent (0–60 minutes)			0.02 (0.01)*** [1.01–1.03]	0.02 (0.01)*** [1.01–1.03]	0.02 (0.01)*** [1.01–1.03]
Current age (18–75 years)				-0.00 (0.01) [0.99–1.01]	-0.00 (0.00) [0.99–1.01]
Education (1–5)				0.10 (0.07) [0.97–1.27]	0.08 (0.07) [0.95–1.25]
Religiosity (1–7)				0.14 (0.06)** [1.04–1.28]	0.13 (0.06)* [1.01–1.26]
Sex (0 = female, 1 = male)					-0.47 (0.15)** [0.46–0.84]
Rightwing political attitude (1–9)					-0.09 (0.04)* [0.84–0.99]
Cox & Snell Pseudo R^2^	12.4%	14.2%	15.8%	16.7%	18.2%

Note

*** = *p* < .001

** = *p* < .01

* = *p* < .05

#### Donation experience

Model 1 showed that whereas profoundness-receptivity predicted a higher likelihood of being a donor (*p* < .001), bullshit-receptivity predicted a lower likelihood of being a donor (*p* = .003, see Table 3). This suggests that bullshit-sensitivity (i.e. the ability to distinguish pseudo-profound sentences from actually profound sentences) predicts donation experience.

When including all control variables in Model 5, profoundness-receptivity remained significantly positively associated (*p* = .001), whereas bullshit-receptivity was non-significantly negatively associated (*p* = .139), with donation experience. Being female (*p* = .008), older (*p* < .001) and more religious (*p* < .001), as well as having completed a higher education (*p* = .012) and spending more time completing the questionnaire (*p* = .030) were also positively associated with donation experience (see Table 3).

#### Volunteering decision

Model 1 showed that whereas profoundness-receptivity predicted a higher likelihood of volunteering for charity (*p* < .001), bullshit-receptivity predicted a lower likelihood of doing so (*p* < .001, see Table 4). This suggests that bullshit-sensitivity predicts volunteering decisions.

When including all control variables in Model 5, profoundness-receptivity remained significantly positively associated (*p* < .001), and bullshit-receptivity remained significantly negatively associated (*p* < .001). Being female (*p* = .002), more religious (*p* = .027) and leaning to the left politically (*p* = .023), as well as having a higher cognitive ability (*p* < .001) and spending a longer time completing the survey (*p* < .001) were also associated with responding YES on the volunteering decision.

## Discussion

This study is the first to demonstrate that individual differences in how people react to pseudo-profound bullshit statements and to actually profound statements predict their behavior. People with high *bullshit-receptivity* (i.e. those who find pseudo-profound bullshit statements such as “the unexplainable touches on the inherent experiences of the universe” to be highly meaningful) were overall less likely to engage in prosocial behavior than people with low bullshit-receptivity. Conversely, people with high *profoundness-receptivity* (i.e. those who think that actually profound statements such as “your teacher can open the door, but you have to step in” are highly meaningful), were overall more likely to engage in prosocial behavior than those with low profoundness-receptivity.

This pattern emerged both when prosocial behavior was assessed in terms of participants’ self-reported donation experience (whether or not they have donated to charity in the past year) and, even clearer, when it was measured in terms of participants’ likelihood to volunteer in order to raise money to charity. For the most part, it also held up well when controlling for potential intermediating factors such as cognitive ability, education, religiosity, age, and time spent completing the survey. In addition to this, the fact that we had over a thousand participants from a roughly nationally representative Swedish sample, gives us further reason to think that the obtained relations between bullshit- and profoundness-receptivity and prosocial behavior are robust and generalizable.

The take-home message of this article is that although bullshit-receptivity and profoundness-receptivity were positively correlated with each other, they yet correlated in opposite directions with prosocial behavior, which suggests that it is primarily individual differences in *bullshit-sensitivity* (the ability to distinguish the pseudo-profound from the actually profound), and not individual differences in the propensity to perceive any given sentence as meaningful, that positively predict prosocial behavior.

The obtained results supports Pennycook et al.’s [1] assertion that individual differences in how people react and respond to bullshit is a relevant and important construct that tells us something about a person over and above her cognitive ability (see also [32]). However, the results also show that future research on the psychology of bullshit needs to consider not just people’s receptivity to bullshit *per se*, but that it should situate bullshit-receptivity in the context of people’s ability to distinguish bullshit from the actually profound.

In addition to our main findings, the current study tested whether results from previous research (exclusively studying Mechanical Turk users or US undergraduate students) held up if tested on a heterogeneous and non-American sample. Generally, in line with the findings of Pennycook et al. [1], we found that religiosity correlated positively with a tendency to perceive meaningfulness in bullshit sentences. Also consistent with previous studies, cognitive ability (numeracy and reflective thinking as measured with the CRT) correlated negatively with bullshit-receptivity but positively with profoundness-receptivity. Participants’ age and level of education also correlated negatively with bullshit-receptivity but positively with profoundness-receptivity, even when controlling for the shared variance. However, unlike previous research conducted in the US [6, 7], we did not find any strong and consistent relation between reactions to bullshit and political self-placement on a left-right scale in the Swedish context (but see [28] for a more detailed discussion about the complex relation between bullshit and political ideology).

### Limitations

Although we supplemented the regression analyses with structural equation modeling (reported in S5 File (Appendix A)) to account for measurement error, no statistical techniques can address validity issues. Some demographic factors, such as sex and age, are unproblematic in terms of validity, because most people know, and are willing to report, their sex and age. But time spent completing the survey is a very indirect (albeit precise) measure of effort, and our measures of religiosity, political attitudes and cognitive ability also leave some room for improvement. Further studies are needed to closely scrutinize the relation between bullshit-sensitivity and prosocial behavior with more rigorous controls in place. Another weakness of the current study is obviously that it was correlational, which makes it impossible to make well-grounded inferences about causation. Additionally, although we believe that the current study contributes to the field by being one of the first to investigate bullshit-sensitivity in a non-American heterogeneous sample, we are very aware that Sweden is just another characteristically WEIRD (i.e. Western, Educated, Industrialized, Rich and Democratic) country [26]. Future studies could test the cultural generalizability of the obtained findings.

More generally, the obtained results were admittedly discovered in post hoc analyses and despite the clarity of our findings, the explanation of *why* highly bullshit-sensitive people behave more prosocial is far from straightforward. Some variable that we did not measure might explain this relation and at this point we do not know which one. One possibility is that bullshit-sensitivity could reflect a general propensity to engage in a global form of critical thinking, including both critically examining information and critically examining the current state of affairs in the world. This is consistent with the notion that more thoughtful and reflective processes will make people adhere to their moral and professional obligations to a greater extent [15]. As an illustration of this explanation, take the influential contemporary philosopher Peter Singer. Singer stands out as both exceptionally good at distinguishing the pseudo-profound from the actually profound (given that he is a scholar in analytical philosophy), and at the same time remarkably motivated to make the world a better place both by helping others and by persuading others to help more [33]. It is conceivable that some of the variation in people’s prosocial behavior is explained not by dispositional empathy, but rather by a general, existentially reflective “Singer-ian” motivation to critically examine the current state of affairs and correct inconsistencies both outside and inside oneself, and consequently to help primarily because of a perceived obligation to do so. If this explanation is correct, persons who lack this type of critical thinking are more inclined to take their relative privileged position in the world for granted and not contemplate whether or not they deserve it or whether or not they have an obligation to help others who are worse off (that is, to engage in epistemically motivated system justification [34])–whereas those who think critically are inclined to evaluate and question the fairness and ethicality of the existing status quo and therefore more likely to do something about it.

There are of course alternative explanations. Both bullshit-sensitivity and prosocial behavior might be reflections of a common underlying personality factor. Some personality researchers [35, 36] have suggested that the covariation between the Big Five personality traits can be explained in terms of a general (“Big One”) personality factor that encompasses a range of socially desirable traits, such as altruism, openness, and conscientiousness. Also, it might be individual differences in self-control and patience [22], rather than critical thinking, that explain the relation between bullshit-sensitivity and prosocial behavior. Self-control has been linked both to cognitive abilities [12, 37] and to prosocial behavior in public goods games [38, 39], and could therefore be relevant in this context.

### Future directions

Future studies should either test whether other mechanisms (such as the ones suggested above) can explain the positive relation between bullshit-sensitivity and prosocial behavior, or test whether increases in bullshit-sensitivity (either natural or experimentally induced) lead to increases in prosocial behavior.

The most straightforward way to make people more bullshit-sensitive is arguably to teach them critical thinking. One could thus randomly allocate participants into two groups that take a course in critical thinking during two different semesters, and assess how both groups’ bullshit-sensitivity and especially prosocial behavior change over time. If prosocial behavior is boosted specifically during (or soon after) the course in critical thinking takes place, and this effect is mediated by increases in bullshit-sensitivity, this would support the idea that bullshit-sensitivity increases prosocial behavior–and have substantial practical implications when it comes to how we should educate our children.

## Supporting information

S1 File Summary of each section included in the survey (see S2 File for an English translation of the full survey). (DOCX)

S2 File English translation and Swedish original of the full survey. (DOCX)

S3 File Key for understanding variables in dataset. (DOCX)

S4 File Raw data in .csv format. (XLS)

S5 File **Appendix A: Structural Equation Modelling**.
(DOCX)
