# Supplementary material for: Bullshit-sensitivity predicts prosocial behavior
Source: PLoS One. 2018 Jul 31;13(7):e0201474. doi: 10.1371/journal.pone.0201474 (PMC6067753; doi:10.1371/journal.pone.0201474)
Supplement: S1 File — (DOCX) [file pone.0201474.s001.docx]

**Supplementary Information 1.**

Summary of each section included in the survey. See Supplementary Material 2 for an English translation of the full survey.

| Survey introduction |  |
| --- | --- |
| ↓ |  |
| **Section 1** | Participants read a text about a research study and were asked to state which conclusion was supported. Participants were randomly allocated to read either about a neutral topic (skin rash) or about a politically loaded topic (immigrant criminality). Results from this section are presented in a separate manuscript [29]. |
| ↓ |  |
| **Section 2** | Four questions about how participants see themselves in terms of traditional and modern ideological labels. Results involving these scales are presented in [28]   1. Do you personally believe that economic equality or economic freedom is relatively more important? 2. Do you describe yourself as relatively more conservative or liberal in social questions? 3. Do you describe yourself as a “World-citizen” or as a “Swede”? 4. Do you describe yourself as someone finding free speech or concern for not hurting the feelings of others more important? |
| ↓ |  |
| **Section 3** | The Moral Foundations Questionnaire (30 items, available in full from *http://moralfoundations.org/questionnaires*). Between the moral foundations judgment items and the moral foundations relevance items were questions measuring just-world belief (8 items), religious beliefs (2 items), religious identity (1 item), spiritual universalism (2 items), , preference for equality (4 items), and resistance to change (4 items). Results involving these scales are presented in [27]. |
| ↓ |  |
| **Section 4** | Three numeracy questions followed by three CRT questions (open-ended)   1. In a small American lottery the chance of winning 10 dollars is 1%. How many do you guess will win 10 dollars if 1000 persons by one ticket each? 2. Imagine that we throw a five-sided die 50 times. How many of these 50 throws will this die eventually land on an uneven number (1, 3 or 5)? 3. Out of 1000 people in a town, 500 are members of a choir. Out of these 500 choir-members, 100 are men. Out of the 500 people not members of a choir, 300 are men. What is the probability that a randomly drawn man is a member of a choir? 4. A bat and a ball cost $110 in total. The bat costs $100 more than the ball. How much does the ball cost? 5. If it takes 5 machines 5 minutes to make 5 widgets, how long would it take 100 machines to make 100 widgets? 6. In a lake, there is a patch of lily pads. Every day, the patch doubles in size. If it takes 48 days for the patch to cover the entire lake, how long would it take for the patch to cover half of the lake? |
| ↓ |  |
| **Section 5** | Bullshit-receptivity and profoundness-receptivity (see list in Table 1) |
| ↓ |  |
| **Section 6** | Participants read and responded to three hypothetical moral dilemmas where they were forced to prioritize among two suggested helping projects. Participants were randomly allocated to respond to how they *would* choose, how they *should* choose or what would be the *moral choice.* Results from this section are used in a separate manuscript currently in preparation. |
| ↓ |  |
| **Section 7** | Similar task as in Section 1. Participants doing the task in Section 1 in a neutral context in Section 1 did it in the loaded context in Section 7 and vice versa. |
| ↓ |  |
| **Section 8** | Mood check 1: Participants rated how happy, sad, and irritated they felt at this specific moment. Results from this section will be used in a separate manuscript. |
| ↓ |  |
| **Section 9** | Volunteering decision question |
| ↓ |  |
| **Section 10** | Mood check 2: Same as in Section 8 |
| ↓ |  |
| **Section 11** | Demographics: Participants responded to the following questions in this order   1. Sex 2. Current age 3. Highest completed education 4. Frequency of religious activities 5. Donation experience question 6. Perceived importance of 17 charitable causes rated on a scale ranging from 1 = *not at all important to me* to 5 = *very important to me*. 7. Have you during the past year given money or in any other way helped begging EU-migrants 8. Do you think it should be legal to beg in Sweden? 9. Political self-placement: “Do you perceive yourself as politically to the left or to the right” 10. If there was an election today, which party would you vote for? Results involving this variable are presented in [28] |
