# Supplementary material for: Bullshit-sensitivity predicts prosocial behavior
Source: PLoS One. 2018 Jul 31;13(7):e0201474. doi: 10.1371/journal.pone.0201474 (PMC6067753; doi:10.1371/journal.pone.0201474)
Supplement: S2 File — (DOCX) [file pone.0201474.s002.docx]

**Supplementary Material 2.**

Survey, December 2016
Swedish original and English translation

| Undersökning om åsikter, attityder och personlighet  Survey about opinions, attitudes and personality  Målet med denna enkät är att undersöka människors åsikter, attityder, personlighet, samt hur dessa saker relaterar till prestation, världsbilder, och beslutsfattande.  Denna undersökning skickas ut till cirka 1000 svenskar och du är en av dessa. Dina svar kommer aldrig att kopplas till dig som individ och som forskare är vi intresserade av din ärliga åsikt så försök att svara helt uppriktigt på alla frågor. Se detta som en chans för dig att säga vad du verkligen tycker, känner och tänker i olika frågor.  Du svarar på frågorna i enkäten genom att markera den siffra som bäst representerar ditt svar.  Stort tack för din medverkan!  The aim of this survey is to investigate people’s opinions, attitudes, and personalities as well as how these things relate to performance, worldviews and decision making.  This survey is sent out to approximately 1000 Swedes and you are one of those. Your responses will never be linked to as an individual and as researchers we are interested in your honest opinion so try to respond sincerely on all questions. See this as an opportunity to say what you really think, feel and believe on different issues.  You respond to the questions by marking the number that best represent your answer  Thank you very much for participating. |
| --- |

**Section 1:**

**25% read version 1a, 25% read version 1b, 25% read version 2a, 25% read version 2b.**

| **1a.**  **I denna del så är din uppgift att läsa om en studie och därefter svara på vilken slutsats som får stöd av resultaten i studien. Läs texten noga och svara genom att markera det alternativ som bäst motsvarar ditt svar.**  **Forskare har framställt en ny kräm för att behandla ett specifikt hudbesvär. Nya behandlingar fungerar ibland men andra gånger gör de istället besvären värre. Dessutom, även i de fall krämen inte har någon effekt, så händer det att besvären minskar eller ökar av andra orsaker. Det är därför nödvändigt att testa alla nya krämer i kontrollerade studier för att se om de på det stora hela minskar eller ökar hudbesvären.**  **Forskare har utfört en studie på patienter med det specifika hudbesväret. I denna studie fick en grupp patienter använda den nya krämen under två veckors tid medan en annan grupp patienter inte använde den nya krämen.**  **Antalet patienter vars hudbesvär blev bättre och antalet patienter vars hudbesvär blev sämre i de respektive grupperna visas i tabellen nedan. Det totala antalet patienter i de båda grupperna är inte lika stort, men det är fortfarande möjligt att bedöma hur effektiv den nya krämen är.**  **Din uppgift är att bedöma om studien visar att det är troligt att patienter som använder den nya krämen får minskade eller ökade hudbesvär jämfört med de patienter som inte använder den nya krämen.**  **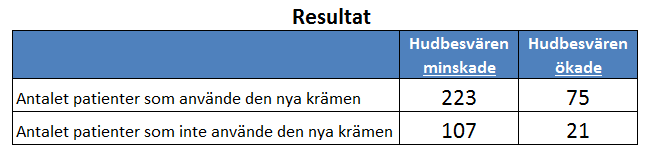**  **In this section your task is to read about a study and thereafter respond which conclusion that is supported by the results in the study. Read the text carefully and respond by marking the alternative that best represent your answer.**  **Researchers have developed a new lotion for treating a specific skin rash. New treatments sometimes work but other times they make the problems worse. Also, even in those cases where the cream have no effect, the skin rash sometimes decrease or increase for different reasons. It is therefore necessary to test all new lotions in controlled studies to see if they decrease or increase skin rash overall.**  **Researcher have conducted a study on patients with the specific skin rash. In this study, one group of patients used the new lotion for a two week period whereas another group did not use the new lotion.**  **The number of patients who had decreased skin rash problems and the number of patients who had increasing skin rash problems in the respective groups can be seen in the table below. The total number of patients in the groups are not the same, but it is still possible to evaluate how effective the new lotion is.**  **Your task is to evaluate if the study shows that it is likely that patients using the new lotion will get decreasing or increasing skin rash problems compared to the patients not using the new lotion.**  **Results**   \|  \| **Skin rash problems decreased** \| **Skin rash problems increased** \| \| --- \| --- \| --- \| \| **Number of patients using the new lotion** \| **223** \| **75** \| \| **Number of patients not using the new lotion** \| **107** \| **21** \|     **Vilken slutsats får stöd av studien?**  **Which conclusion is supported in this study?** |
| --- | --- | --- | --- | --- | --- | --- | --- | --- | --- |

| ❑ | Det är troligt att patienter som använder den nya krämen får minskade hudbesvär jämfört med de patienter som inte använder den nya krämen.  It is likely that patients using the new lotion will have decreased skin rash problems compared to patients not using the new lotion |
| --- | --- |
| ❑ | Det är troligt att patienter som använder den nya krämen får ökade hudbesvär jämfört med de patienter som inte använder den nya krämen.  It is likely that patients using the new lotion will have increased skin rash problems compared to patients not using the new lotion  -------------------------------------Sidbrytning-------------------------------------------------------- |

| **1b.**  **I denna del så är din uppgift att läsa om en studie och därefter svara på vilken slutsats som får stöd av resultaten i studien. Läs texten noga och svara genom att markera det alternativ som bäst motsvarar ditt svar.**  **Forskare har framställt en ny kräm för att behandla ett specifikt hudbesvär. Nya behandlingar fungerar ibland men andra gånger gör de istället besvären värre. Dessutom, även i de fall krämen inte har någon effekt, så händer det att besvären minskar eller ökar av andra orsaker. Det är därför nödvändigt att testa alla nya krämer i kontrollerade studier för att se om de på det stora hela minskar eller ökar hudbesvären.**  **Forskare har utfört en studie på patienter med det specifika hudbesväret. I denna studie fick en grupp patienter använda den nya krämen under två veckors tid medan en annan grupp patienter inte använde den nya krämen.**  **Antalet patienter vars hudbesvär blev bättre och antalet patienter vars hudbesvär blev sämre i de respektive grupperna visas i tabellen nedan. Det totala antalet patienter i de båda grupperna är inte lika stort, men det är fortfarande möjligt att bedöma hur effektiv den nya krämen är.**  **Din uppgift är att bedöma om studien visar att det är troligt att patienter som använder den nya krämen får minskade eller ökade hudbesvär jämfört med de patienter som inte använder den nya krämen.**    **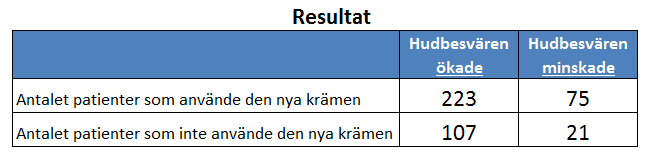**  **In this section your task is to read about a study and thereafter respond which conclusion that is supported by the results in the study. Read the text carefully and respond by marking the alternative that best represent your answer**  **Researchers have developed a new lotion for treating a specific skin rash. New treatments sometimes work but other times they make the problems worse. Also, even in those cases where the cream have no effect, the skin rash sometimes decrease or increase for different reasons. It is therefore necessary to test all new lotions in controlled studies to see if they decrease or increase skin rash overall.**  **Researcher have conducted a study on patients with the specific skin rash. In this study, one group of patients used the new lotion for a two week period whereas another group did not use the new lotion.**  **The number of patients who had decreased skin rash problems and the number of patients who had increasing skin rash problems in the respective groups can be seen in the table below. The total number of patients in the groups are not the same, but it is still possible to evaluate how effective the new lotion is.**  **Your task is to evaluate if the study shows that it is likely that patients using the new lotion will get decreasing or increasing skin rash problems compared to the patients not using the new lotion.**  **Results**   \|  \| **Skin rash problems increased** \| **Skin rash problems decreased** \| \| --- \| --- \| --- \| \| **Number of patients using the new lotion** \| **223** \| **75** \| \| **Number of patients not using the new lotion** \| **107** \| **21** \|   **Vilken slutsats får stöd av studien?**  **Which conclusion is supported in this study?** |
| --- | --- | --- | --- | --- | --- | --- | --- | --- | --- |

| ❑ | Det är troligt att patienter som använder den nya krämen får minskade hudbesvär jämfört med de patienter som inte använder den nya krämen.  It is likely that patients using the new lotion will have decreased skin rash problems compared to patients not using the new lotion |
| --- | --- |
| ❑ | Det är troligt att patienter som använder den nya krämen får ökade hudbesvär jämfört med de patienter som inte använder den nya krämen.  It is likely that patients using the new lotion will have increased skin rash problems compared to patients not using the new lotion  -------------------------------------Sidbrytning-------------------------------------------------------- |

-------------------------------------Sidbrytning--------------------------------------------------------

| **2a.**  **I denna del så är din uppgift att läsa om en studie och därefter svara på vilken slutsats som får stöd av resultaten i studien. Läs texten noga och svara genom att markera det alternativ som bäst motsvarar ditt svar.**  **Vissa små norska samhällen tar emot flyktingar medan andra inte gör det. Ibland ökar den totala kriminaliteten när flyktingar kommer, och ibland minskar den. Dessutom, även i samhällen som inte tar emot flyktingar så kan kriminaliteten minska eller öka av andra orsaker. Det är därför nödvändigt att testa vilken effekt flyktingmottagande har på kriminaliteten i kontrollerade studier för att se om mottagandet av flyktingar på det stora hela minskar eller ökar kriminaliteten.**  **Forskare i Norge har undersökt hur kriminaliteten ändrats under de senaste fem åren i små norska samhällen som antingen inte har tagit emot flyktingar eller som har tagit emot flyktingar under denna period.**  **Antalet norska samhällen med/utan flyktingmottagande där kriminaliteten minskade och antalet norska samhällen med/utan flyktingmottagande där kriminaliteten ökade visas i tabellen nedan. Det totala antalet samhällen med/utan flyktingar är inte lika stort, men det är fortfarande möjligt att bedöma hur flyktingmottagande och kriminalitet är relaterade.**  **Din uppgift är att bedöma om studien visar att det är troligt att kriminaliteten har ökat eller minskat i norska samhällen som tagit emot flyktingar, jämfört med i norska samhällen som inte gjort det.**  **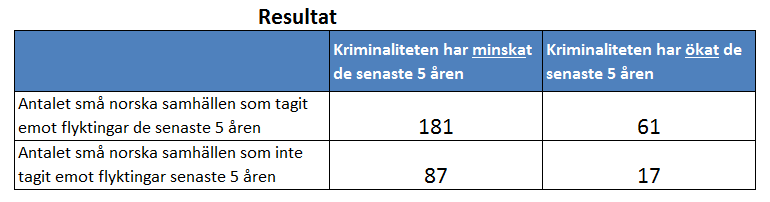**  **In this section your task is to read about a study and thereafter respond which conclusion that is supported by the results in the study. Read the text carefully and respond by marking the alternative that best represent your answer**  **Some Norwegian villages accepts refugees whereas other do not. Sometimes the total criminality rate increase when refugees arrive, and at other times it decrease. Also, in villages that does not accept refuges, the criminality rate can decrease or increase for different reasons. It is therefore necessary to use controlled studies for testing what effect accepting refugees has on the criminality rate.**  **Researchers in Norway have investigated how the criminality rate changed in the past five years in small Norwegian villages that either did not accept or accepted refugees during this period.**  **The number of Norwegian villages accepting/not accepting refugees where the criminality rate decreased and the number of Norwegian villages accepting/not accepting refugees where the criminality rate increased are shown in the table below. The total number of villages accepting/not accepting refugees are not the same, but it is still possible to evaluate how acceptance of refugees and criminality rate are related.**  **Your task is to evaluate if the study shows that it is likely that the criminality rate has increased or decreased in Norwegian villages that accepted refugees, compared to Norwegian villages that did not accept refugees.**  **Results**   \|  \| **Criminality rate decreased the past 5 years** \| **Criminality rate increased  the past 5 years** \| \| --- \| --- \| --- \| \| **Number of Norwegian villages accepting refugees the past 5 years** \| **181** \| **61** \| \| **Number of Norwegian villages not accepting refugees the past 5 years** \| **87** \| **17** \|   **Vilken slutsats får stöd av studien?**  **Which conclusion is supported in this study?** |
| --- | --- | --- | --- | --- | --- | --- | --- | --- | --- |

| ❑ | Det är troligt att kriminaliteten har minskat de senaste 5 åren i norska samhällen som tagit emot flyktingar jämfört med i norska samhällen som inte gjort det.  It is likely that the criminality rate decreased in the past 5 years, in Norwegian villages that accepted refugees compared to Norwegian villages that did not accept refugees. |
| --- | --- |
| ❑ | Det är troligt att kriminaliteten har ökat de senaste 5 åren i norska samhällen som tagit emot flyktingar jämfört med i norska samhällen som inte gjort det.  It is likely that the criminality rate increased in the past 5 years, in Norwegian villages that accepted refugees compared to Norwegian villages that did not accept refugees. |

-------------------------------------Sidbrytning--------------------------------------------------------

| **2b.**  **I denna del så är din uppgift att läsa om en studie och därefter svara på vilken slutsats som får stöd av resultaten i studien. Läs texten noga och svara genom att markera det alternativ som bäst motsvarar ditt svar.**  **Vissa små norska samhällen tar emot flyktingar medan andra inte gör det. Ibland ökar den totala kriminaliteten när flyktingar kommer, och ibland minskar den. Dessutom, även i samhällen som inte tar emot flyktingar så kan kriminaliteten minska eller öka av andra orsaker. Det är därför nödvändigt att testa vilken effekt flyktingmottagande har på kriminaliteten i kontrollerade studier för att se om mottagandet av flyktingar på det stora hela minskar eller ökar kriminaliteten.**  **Forskare i Norge har undersökt hur kriminaliteten ändrats under de senaste fem åren i små norska samhällen som antingen inte har tagit emot flyktingar eller som har tagit emot flyktingar under denna period.**  **Antalet norska samhällen med/utan flyktingmottagande där kriminaliteten minskade och antalet norska samhällen med/utan flyktingmottagande där kriminaliteten ökade visas i tabellen nedan. Det totala antalet samhällen med/utan flyktingar är inte lika stort, men det är fortfarande möjligt att bedöma hur flyktingmottagande och kriminalitet är relaterade.**  **Din uppgift är att bedöma om studien visar att det är troligt att kriminaliteten har ökat eller minskat i norska samhällen som tagit emot flyktingar, jämfört med i norska samhällen som inte gjort det.**  **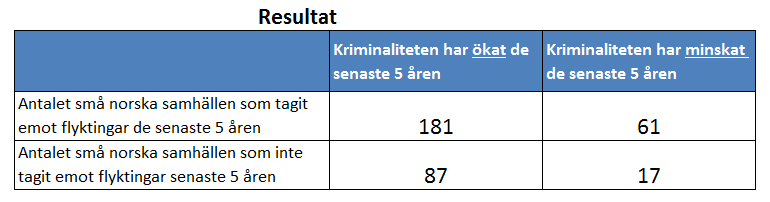**  **In this section your task is to read about a study and thereafter respond which conclusion that is supported by the results in the study. Read the text carefully and respond by marking the alternative that best represent your answer**  **Some Norwegian villages accepts refugees whereas other do not. Sometimes the total criminality rate increase when refugees arrive, and at other times it decrease. Also, in villages that does not accept refuges, the criminality rate can decrease or increase for different reasons. It is therefore necessary to use controlled studies for testing what effect accepting refugees has on the criminality rate.**  **Researchers in Norway have investigated how the criminality rate changed in the past five years in small Norwegian villages that either did not accept or accepted refugees during this period.**  **The number of Norwegian villages accepting/not accepting refugees where the criminality rate decreased and the number of Norwegian villages accepting/not accepting refugees where the criminality rate increased are shown in the table below. The total number of villages accepting/not accepting refugees are not the same, but it is still possible to evaluate how acceptance of refugees and criminality rate are related.**  **Your task is to evaluate if the study shows that it is likely that the criminality rate has increased or decreased in Norwegian villages that accepted refugees, compared to Norwegian villages that did not accept refugees.**  **Results**   \|  \| **Criminality rate increased the past 5 years** \| **Criminality rate decreased  the past 5 years** \| \| --- \| --- \| --- \| \| **Number of Norwegian villages accepting refugees the past 5 years** \| **181** \| **61** \| \| **Number of Norwegian villages not accepting refugees the past 5 years** \| **87** \| **17** \|   **Vilken slutsats får stöd av studien?** |
| --- | --- | --- | --- | --- | --- | --- | --- | --- | --- |

| ❑ | Det är troligt att kriminaliteten har minskat de senaste 5 åren i norska samhällen som tagit emot flyktingar jämfört med i norska samhällen som inte gjort det.  It is likely that the criminality rate decreased in the past 5 years, in Norwegian villages that accepted refugees compared to Norwegian villages that did not accept refugees. |
| --- | --- |
| ❑ | Det är troligt att kriminaliteten har ökat de senaste 5 åren i norska samhällen som tagit emot flyktingar jämfört med i norska samhällen som inte gjort det.  It is likely that the criminality rate increased in the past 5 years, in Norwegian villages that accepted refugees compared to Norwegian villages that did not accept refugees. |

--------------------------------------------Sidbrytning----------------------------------------------------------------------

**Section 2**

| **Nästa del handlar om dina åsikter och attityder. Varje fråga börjar med att två begrepp definieras. Utgå från definitionerna som ges i frågan. Läs varje fråga noga och svara genom att markera det alternativ som bäst representerar ditt svar.**  **The next section is about your opinions and attitudes. Each questions begins with a definition of two concepts. Please base your responses on the definitions given in the questions. Read each question carefully and mark the alternative that best represent your answer.**  **The order of questions A-D are randomized.** |
| --- |

-------------------------------------Sidbrytning--------------------------------------------------------

| **A**  **Ibland pratar man om huruvida människor prioriterar ekonomisk jämlikhet eller ekonomisk frihet.**  **Människor som prioriterar ekonomisk jämlikhet i ett samhälle, menar att det är viktigt att minska de ekonomiska klyftorna i samhället även om det leder till att ekonomiska resurser omfördelas från de som har mycket till de som har lite.**  **Människor som prioriterar värdet av ekonomisk frihet menar att varje individ har rätt till resultaten av sin ekonomiska framgång, även om det leder till ekonomiska ojämlikheter i samhället.**  **Om man använder definitionen ovan, tycker du personligen att ekonomisk jämlikhet eller ekonomisk frihet är det relativt viktigare?**  **Sometimes we talk about people prioritizing economic equality or economic freedom. People prioritizing economic equality in a society, think that it is important to decrease the economic gaps in the society even if that leads to economic resources being transferred from those having much to those having less.  People who prioritize the value of economic freedom think that each individual have the right to the results of his/her economic success, even if that will lead to greater inequalities in the society.**  **If using the definition above, do you personally believe that economic equality or economic freedom are the relatively more important?** |
| --- |

|  | Jag tycker att ekonomisk jämlikhet är mycket viktigare  I think economic equality is much more important | Jag tycker att ekonomisk jämlikhet är viktigare  I think economic equality is more important | Jag tycker att ekonomisk jämlikhet är lite viktigare I think economic equality is slightly more important | Jag tycker båda är lika viktiga/ lika oviktiga I think both are equally important/ equally unimportant | Jag tycker att ekonomisk frihet är lite viktigare  I think economic freedom is slightly more important | Jag tycker att ekonomisk frihet är viktigare I think economic freedom is more important | Jag tycker att ekonomisk frihet är mycket viktigare I think economic freedom is much more important |
| --- | --- | --- | --- | --- | --- | --- | --- |
|  | ❑ | ❑ | ❑ | ❑ | ❑ | ❑ | ❑ |

-------------------------------------Sidbrytning--------------------------------------------------------

| **B**  **Ibland pratar man om hur konservativ eller liberal en person är i sociala frågor.**  **Människor som är socialt konservativa betonar värdet av samhällets traditioner och av att ibland rätta in sig i ledet och följa de normer som finns.**  **Människor som är socialt liberala betonar värdet av den individuella friheten och alla individers rättighet att kunna vara sig själva fullt ut.**  **Om man använder definitionen ovan, skulle du beskriva dig själv som relativt mer konservativ eller relativt mer liberal i sociala frågor?**  **Sometimes we speak about how conservative or liberal a person is concerning social issues.  People who are socially conservative emphasize the value of societal traditions and of at times conforming to the public opinion and following the existing norms. People who are socially liberal emphasize the value of the individual freedom and all individual’s right to be oneself to a full extent.**  **If using the definition above, would you describe yourself as relatively more conservative or relatively more liberal regarding social issues?** |
| --- |

|  | Jag lutar mycket mer åt det konservativa hållet  I lean much more toward conservatism | Jag lutar åt det konservativa hållet I lean toward conservatism | Jag lutar svagt åt det konservativa hållet  I lean slightly toward conservatism | Jag är i mitten/ jag är varken konservativ eller liberal I am in the middle/neither conservative nor liberal | Jag lutar svagt åt det liberala hållet I lean slightly toward liberalism | Jag lutar åt det liberala hållet I lean toward liberalism | Jag lutar mycket mer åt det liberala hållet I lean much more toward liberalism |
| --- | --- | --- | --- | --- | --- | --- | --- |
|  | ❑ | ❑ | ❑ | ❑ | ❑ | ❑ | ❑ |

-------------------------------------Sidbrytning--------------------------------------------------------

| **C**  **Ibland pratar man om huruvida människor ser sig själva som ”Världsmedborgare” eller som ”Svenskar”.**  **Människor som ser sig som ”Världsmedborgare” menar att alla människor bör ha samma rättigheter, och att vi har ett lika stort ansvar att hjälpa alla människor, oavsett deras nationalitet.**  **Människor som ser sig som ”Svenskar” menar att svenska medborgare bör ha vissa privilegier, och att vi har ett större ansvar att hjälpa andra svenskar än vad vi har att hjälpa människor från andra länder.**  **Om man använder definitionen ovan, skulle du beskriva dig själv mer som en ”Världsmedborgare” eller mer som en ”Svensk”?**  **Sometimes we speak about whether people identify as ”World citizens” or as ”Swedes”.**  **People identifying as “World citizens” mean that all people should have the same rights and that we have an equally great responsibility to help all people, no matter their nationality.**  **People identifying as “Swedes” mean that Swedish citizens should have certain privileges, and that we have a greater responsibility to help other Swedes than we have to help people from other countries.**  **If using the definition above, would you describe yourself more as a “World citizen” or more as a “Swede”** |
| --- |

|  | Jag är mycket  mer av en ”Världs-  medborgare” I am much more a ”World citizen” | Jag är mer av en ”Världs-  medborgare” I am more a ”World citizen” | Jag är lite mer av en ”Världs-  Medborgare I am slightly more a World Citizen | Jag är lika mycket/ lika lite en ”Världs-  medborgare” som en ”Svensk”  I am equally much / little a world citizen as a Swede | Jag är lite mer av en ”Svensk” I am slightly more a Swede | Jag är mer av en ”Svensk” I am more a ”Swede | Jag är mycket mer av en ”Svensk” I am much more a ”Swede” |
| --- | --- | --- | --- | --- | --- | --- | --- |
|  | ❑ | ❑ | ❑ | ❑ | ❑ | ❑ | ❑ |

-------------------------------------Sidbrytning--------------------------------------------------------

| **D**  **Ibland pratar man om hur människor ser på värdet av det fria ordet, och på värdet av hänsyn.**  **Människor som betonar värdet av det fria ordet menar att det bör vara ”högt i tak” och att alla bör kunna uttrycka sin åsikt öppet, oavsett hur obekväm åsikten är, och även om den sticker i ögonen.**  **Människor som betonar värdet av hänsyn menar att det är oacceptabelt att uttrycka sig provocerande och att det bör vara olagligt att använda ord eller uttryck som upplevs kränkande.**  **Om man använder definitionen ovan, skulle du beskriva dig själv som en som tycker det fria ordet är det relativt viktigare eller en som tycker att hänsyn är det relativt viktigare?**  **Sometimes we speak about how people perceive the value of free speech and the value of concern for others feelings. People who emphasize the value of the free speech mean that it should be an “open discussion climate” and that everyone should be able to express his/her opinion, even if that opinion is uncomfortable and even if it sticks is some people’s eyes.**  **People who emphasize the value of concern for others feelings mean that it is unacceptable to express in a way that is provoking and that it should be illegal to use words or expressions that is perceived as offending.**  **If using the definition above, would you describe yourself as someone who thinks that free speech as relatively more important or as someone who thinks that concern for others are more important?** |
| --- |

|  | Jag tycker att det fria ordet är mycket viktigare I think free speech is much more important | Jag tycker det fria ordet är viktigare  I think free speech is more important | Jag tycker att det fria ordet är lite viktigare  I think free speech is slightly more important | Jag tycker det fria ordet och hänsyn är lika viktiga/lika oviktiga  I think free speech and concern for others feeling are equally important/unimportant | Jag tycker att hänsyn är lite viktigare I think concern for others feelings is slightly more important | Jag tycker hänsyn är viktigare I think concern for others feelings is more important | Jag tycker att hänsyn är mycket viktigare I think concern for others feelings is much more important |
| --- | --- | --- | --- | --- | --- | --- | --- |
|  | ❑ | ❑ | ❑ | ❑ | ❑ | ❑ | ❑ |

-------------------------------------Sidbrytning--------------------------------------------------------

**Section 3**

| **I denna del kommer du att få läsa ett antal påståenden om världen och samhället. Din uppgift är att markera i vilken grad du håller med om varje påstående.**  **In this section, you will read a number of statements about the world or about the society. Your task is to mark to what extent you agree with each statement.** |
| --- |

**Order of question 1-15 randomized**

| **1-15** |
| --- |

|  | Håller inte alls med  Strongly disagree | Håller till stor del inte med  Moderatedly disagree | Håller delvis i nte med  Slightly disagree | Neutral/  vet ej  Neutral/do no know | Håller delvis med Slightly agree | Håller till stor del med Moderatedly agree | Håller med fullständigt Strongly agree |
| --- | --- | --- | --- | --- | --- | --- | --- |
| Medlidande med dem som lider är den mest avgörande dygden.  Compassion for those who are suffering is the most crucial virtue. | ❑ | ❑ | ❑ | ❑ | ❑ | ❑ | ❑ |
| När de styrande politikerna skapar lagar bör den främsta principen vara att försäkra sig om att alla behandlas rättvist.  When the government makes laws, the number one principle should be ensuring that everyone is treated fairly. | ❑ | ❑ | ❑ | ❑ | ❑ | ❑ | ❑ |
| Jag är stolt över mitt lands historia.  I am proud of my country’s history. | ❑ | ❑ | ❑ | ❑ | ❑ | ❑ | ❑ |
| Respekt för auktoritet är något som alla barn behöver lära sig.  Respect for authority is something all children need to learn. | ❑ | ❑ | ❑ | ❑ | ❑ | ❑ | ❑ |
| Människor bör inte göra saker som är äckliga, även om ingen kommer till skada.  People should not do things that are disgusting, even if no one is harmed. | ❑ | ❑ | ❑ | ❑ | ❑ | ❑ | ❑ |
| En av de värsta saker en person skulle kunna göra är att skada ett försvarslöst djur.  One of the worst things a person could do is hurt a defenseless animal. | ❑ | ❑ | ❑ | ❑ | ❑ | ❑ | ❑ |
| Rättvisa är det viktigaste kravet på ett samhälle. Justice is the most important requirement for a society. | ❑ | ❑ | ❑ | ❑ | ❑ | ❑ | ❑ |
| Människor bör vara lojala mot sina familjemedlemmar även när dessa har gjort något fel.  People should be loyal to their family members, even when they have done something wrong. | ❑ | ❑ | ❑ | ❑ | ❑ | ❑ | ❑ |
| Män och kvinnor har olika roller att spela i samhället.  Men and women each have different roles to play in society. | ❑ | ❑ | ❑ | ❑ | ❑ | ❑ | ❑ |
| Jag skulle kalla vissa handlingar felaktiga baserat på att de är onaturliga. I would call some acts wrong on the grounds that they are unnatural. | ❑ | ❑ | ❑ | ❑ | ❑ | ❑ | ❑ |
| Det kan aldrig vara rätt att döda en människa. It can never be right to kill a human being. | ❑ | ❑ | ❑ | ❑ | ❑ | ❑ | ❑ |
| Jag anser att det är moraliskt fel att rika barn ärver mycket pengar medan fattiga barn inte ärver något alls.  I think it’s morally wrong that rich children inherit a lot of money while poor children inherit nothing. | ❑ | ❑ | ❑ | ❑ | ❑ | ❑ | ❑ |
| Det är viktigare att vara en lagspelare än att uttrycka sig själv.  It is more important to be a team player than to express oneself. | ❑ | ❑ | ❑ | ❑ | ❑ | ❑ | ❑ |
| Om jag var en soldat och inte höll med om min överordnades order så skulle jag lyda ändå eftersom det är min plikt.  If I were a soldier and disagreed with my commanding officer’s orders, I would obey anyway because that is my duty. | ❑ | ❑ | ❑ | ❑ | ❑ | ❑ | ❑ |
| Kyskhet är en viktig och värdefull dygd.  Chastity is an important and valuable virtue. | ❑ | ❑ | ❑ | ❑ | ❑ | ❑ | ❑ |

-------------------------------------Sidbrytning--------------------------------------------------------

| **16-25** |
| --- |

**Order of question 16-23 randomized.**

|  | Håller inte alls med  Strongly disagree | Håller till stor del inte med  Moderatedly disagree | Håller delvis i nte med Slightly disagree | Neutral/  vet ej  Neutral/do no know | Håller delvis med Slightly agree | Håller till stor del med Moderatedly agree | Håller med fullständigt Strongly agree |
| --- | --- | --- | --- | --- | --- | --- | --- |
| Olyckor drabbar sällan hyggliga och anständiga människor. Misfortune is least likely to strike worthy decent people. | ❑ | ❑ | ❑ | ❑ | ❑ | ❑ | ❑ |
| Överlag får bra människor vad de förtjänar i denna värld. Generally, people deserve what they get in this world. | ❑ | ❑ | ❑ | ❑ | ❑ | ❑ | ❑ |
| Människor kommer att uppleva medgång om de är goda. People will experience good fortune if they themselves are good. | ❑ | ❑ | ❑ | ❑ | ❑ | ❑ | ❑ |
| Generellt sett förtjänar människor vad de får i denna värld. By and large, good people get what they deserve in this world. | ❑ | ❑ | ❑ | ❑ | ❑ | ❑ | ❑ |
| Människors motgångar är ett resultat av misstag de har gjort. People’s misfortunes result from mistakes they have made. | ❑ | ❑ | ❑ | ❑ | ❑ | ❑ | ❑ |
| Genom våra handlingar kan vi förhindra att dåliga saker händer oss. Through our actions we can prevent bad things from happening to us. | ❑ | ❑ | ❑ | ❑ | ❑ | ❑ | ❑ |
| Om människor vidtog förebyggande åtgärder skulle de flesta olyckor kunna undvikas. If people took preventive actions, most misfortune could be avoided. | ❑ | ❑ | ❑ | ❑ | ❑ | ❑ | ❑ |
| När dåliga saker händer är det vanligtvis för att människor inte vidtagit nödvändiga åtgärder för att skydda sig själva.  When bad things happen, it is typically because people have not taken the necessary actions to protect themselves. | ❑ | ❑ | ❑ | ❑ | ❑ | ❑ | ❑ |

-------------------------------------Sidbrytning--------------------------------------------------------

| **24-28** |
| --- |

**Order of question 24-28 randomized.**

|  | Håller inte alls med  Strongly disagree | Håller till stor del inte med Moderatedly disagree | Håller delvis i nte med  Slightly disagree | Neutral/  vet ej  Neutral/do no know | Håller delvis med Slightly agree | Håller till stor del med Moderatedly agree | Håller med fullständigt Strongly agree |
| --- | --- | --- | --- | --- | --- | --- | --- |
| Jag känner att vi alla har ett gemensamt band på ett högre plan. I feel that on a higher level all of us share a common bond | ❑ | ❑ | ❑ | ❑ | ❑ | ❑ | ❑ |
| Allt liv är sammanvävt.  All life is interconnected | ❑ | ❑ | ❑ | ❑ | ❑ | ❑ | ❑ |
| Det finns en högre nivå av medvetande och andlighet som binder samman alla människor. There is a higher plane of consciousness or spirituality that binds all people | ❑ | ❑ | ❑ | ❑ | ❑ | ❑ | ❑ |
| Även om enskilda personer kan vara besvärliga känner jag ett känslomässigt band med hela mänskligheten. Although individual people may be difficult, I feel an emotional bond with all of humanity | ❑ | ❑ | ❑ | ❑ | ❑ | ❑ | ❑ |
| Religion är en viktig del av mitt liv  Religion is an important part of my life | ❑ | ❑ | ❑ | ❑ | ❑ | ❑ | ❑ |

-------------------------------------Sidbrytning--------------------------------------------------------

| **29-36** |
| --- |

**Order of question 29-36 randomized.**

|  | Håller inte alls med  Strongly disagree | Håller till stor del inte med  Moderatedly disagree | Håller delvis i nte med  Slightly disagree | Neutral/  vet ej  Neutral/do no know | Håller delvis med Slightly agree | Håller till stor del med Moderatedly agree | Håller med fullständigt Strongly agree |
| --- | --- | --- | --- | --- | --- | --- | --- |
| Om människor behandlades mer jämlikt i vårt land så skulle vi ha färre problem If people were treated more equally in this country we would have many fewer problems | ❑ | ❑ | ❑ | ❑ | ❑ | ❑ | ❑ |
| Det är ett misstag att försöka garantera en jämn fördelning av resurser mellan rika och fattiga.  It is a mistake to try to guarantee an equal distribution of resources between rich and poor | ❑ | ❑ | ❑ | ❑ | ❑ | ❑ | ❑ |
| Vårt samhälle borde göra vad som än krävs för att se till att alla har lika möjligheter att lyckas.  Our society should do whatever is necessary to make sure that everyone has equal opportunities to succeed | ❑ | ❑ | ❑ | ❑ | ❑ | ❑ | ❑ |
| Stora löneskillnader krävs för att motivera människor att jobba tillräckligt hårt för att förbättra ekonomin.  Substantial income differences are needed to motivate people to work hard enough to improve the economy | ❑ | ❑ | ❑ | ❑ | ❑ | ❑ | ❑ |
| Det skulle vara bättre för Sverige om vi fokuserade mer på traditionella familjeband This country would be better off if there were more emphasis on traditional family ties. | ❑ | ❑ | ❑ | ❑ | ❑ | ❑ | ❑ |
| Våra seder och vårt nationella arv är det som har gjort vårt land bra, och vissa människor borde visa mer respekt för dem Our customs and national heritage are the things that have made us great, and certain people should be made to show greater respect for them. | ❑ | ❑ | ❑ | ❑ | ❑ | ❑ | ❑ |
| Om man börjar förändra saker väldigt mycket slutar det ofta med att det blir till det sämre If you start changing things very much, you often end up making them worse. | ❑ | ❑ | ❑ | ❑ | ❑ | ❑ | ❑ |
| Att ändra på saker i samhället (t.ex. stat, religion, företag) är riskabelt så det är bättre att förändra dem långsamt än snabbt Changing any institution (e.g., government, religion, business) is risky, so it is better to change at a slow than a rapid pace. | ❑ | ❑ | ❑ | ❑ | ❑ | ❑ | ❑ |

-------------------------------------Sidbrytning--------------------------------------------------------

| **Svara på i vilken utsträckning följande överväganden är relevanta för ditt tänkande när du avgör om något är rätt eller fel? When you decide whether something is right or wrong, to what extent are the following considerations relevant to your thinking?** |
| --- |

| Var god bedöm varje påstående med hjälp av denna skala:  Please rate each statement using this scale:  Inte alls relevant (Detta övervägande har inget alls att göra med mina bedömningar av rätt och fel) not at all relevant (This consideration has nothing to do with my judgments of right and wrong)  Inte särskilt relevant  not very relevant  Något relevant  slightly relevant  Ganska relevant  somewhat relevant  Mycket relevant  very relevant  Extremt relevant (Detta är en av de viktigaste faktorerna när jag bedömer rätt och fel)  extremely relevant (This is one of the most important factors when I judge right and wrong) |
| --- |

|  | Inte alls  relevant | Inte särskilt  relevant | Något  relevant | Ganska  relevant | Mycket  relevant | Extremt  relevant |
| --- | --- | --- | --- | --- | --- | --- |
| Huruvida någon led emotionellt Whether or not someone suffered emotionally | ❑ | ❑ | ❑ | ❑ | ❑ | ❑ |
| Huruvida några människor behandlades annorlunda än andra Whether or not some people were treated differently than others | ❑ | ❑ | ❑ | ❑ | ❑ | ❑ |
| Huruvida någons handling uppvisade kärlek för hans eller hennes land Whether or not someone’s action showed love for his or her country | ❑ | ❑ | ❑ | ❑ | ❑ | ❑ |
| Huruvida någon visade brist på respekt för auktoritet Whether or not someone showed a lack of respect for authority | ❑ | ❑ | ❑ | ❑ | ❑ | ❑ |
| Huruvida någon bröt mot regler för renhet och anständighet Whether or not someone violated standards of purity and decency | ❑ | ❑ | ❑ | ❑ | ❑ | ❑ |
| Huruvida någon tog hand om någon som var svag eller sårbar Whether or not someone cared for someone weak or vulnerable | ❑ | ❑ | ❑ | ❑ | ❑ | ❑ |
| Huruvida någon agerade orättvist Whether or not someone acted unfairly | ❑ | ❑ | ❑ | ❑ | ❑ | ❑ |
| Huruvida någon gjorde något för att svika sin grupp Whether or not someone did something to betray his or her group | ❑ | ❑ | ❑ | ❑ | ❑ | ❑ |
| Huruvida någon rättade sig efter samhällets traditioner Whether or not someone conformed to the traditions of society | ❑ | ❑ | ❑ | ❑ | ❑ | ❑ |
| Huruvida någon gjorde något äckligt Whether or not someone did something disgusting | ❑ | ❑ | ❑ | ❑ | ❑ | ❑ |
| Huruvida någon var grym Whether or not someone was cruel | ❑ | ❑ | ❑ | ❑ | ❑ | ❑ |
| Huruvida någon förnekades sina rättigheter Whether or not someone was denied his or her rights | ❑ | ❑ | ❑ | ❑ | ❑ | ❑ |
| Huruvida någon uppvisade en brist på lojalitet Whether or not someone showed a lack of loyalty | ❑ | ❑ | ❑ | ❑ | ❑ | ❑ |
| Huruvida en handling orsakade kaos eller oordning Whether or not an action caused chaos or disorder | ❑ | ❑ | ❑ | ❑ | ❑ | ❑ |
| Huruvida någon handlade på ett sätt som gud skulle godkänna Whether or not someone acted in a way that God would approve of | ❑ | ❑ | ❑ | ❑ | ❑ | ❑ |

-------------------------------------Sidbrytning--------------------------------------------------------

**Section 4**

| **I nästa del kommer du att få lösa några problem. För varje fråga, skriv ned ditt svar i det öppna fältet för varje fråga. Om du inte kan räkna ut rätt svar – klicka på ”vet ej”.**  **In the next section you will solve a number of problems. For each question, write your answer in the open box. If you cannot reach the correct answer – click on “Don’t know”** |
| --- |

-------------------------------------Sidbrytning--------------------------------------------------------

| 1. **I ett litet amerikanskt lotteri så är chansen att vinna 10 dollar 1%. Hur många gissar du kommer att vinna 10 dollar om 1000 personer köper varsin biljett till lotteriet?**   In a small American lottery the chance of winning 10 dollars is 1%. How many do you guess will win 10 dollars if 1000 persons by one ticket each? |
| --- |

| ❑ | Antal personer av 1000: ___________  Number of persons out of 1000 |
| --- | --- |
| ❑ | Vet ej  Don’t know |

-------------------------------------Sidbrytning--------------------------------------------------------

| 1. **Tänk dig att vi kastar en femsidig tärning 50 gånger. Av dessa 50 kast, hur många gånger kommer denna femsidiga tärning till slut att visa en udda siffra (1, 3 eller 5)?**   Imagine that we throw a five-sided die 50 times. How many of these 50 throws will this die eventually land on an uneven number (1, 3 or 5)? |
| --- |

| ❑ | Antal av 50 kast: ___________  Number out of 50 throws |
| --- | --- |
| ❑ | Vet ej Don’t know |

-------------------------------------Sidbrytning--------------------------------------------------------

| 1. **Av 1000 människor i en liten stad är 500 medlemmar i en kör. Av dessa 500 medlemmar i en kör är 100 män. Av de 500 invånarna som inte är med i en kör är 300 män. Vad är sannolikheten att en slumpmässigt dragen man är medlem i kören?**   Out of 1000 people in a town, 500 are members of a choir. Out of these 500 choir-members, 100 are men. Out of the 500 people not members of a choir, 300 are men. What is the probability that a randomly drawn man is a member of a choir? |  |
| --- | --- |
| *Ange sannolikheten i procent.* | |

| ❑ | | Sannolikhet i %: ___________ Probability in % |
| --- | --- | --- |
| ❑ | | Vet ej Don’t know |
| 1. **Ett basebollträ och en boll kostar tillsammans 110 dollar. Träet kostar 100 dollar mer än bollen. Vad kostar bollen?**   A bat and a ball cost $110 in total. The bat costs $100 more than the ball. How much does the ball cost? | |  |

| ❑ | Bollens kostnad i dollar: ___________ The cost of the ball in dollars |
| --- | --- |
| ❑ | Vet ej Don’t know |

-------------------------------------Sidbrytning--------------------------------------------------------

| 1. **Om det tar 5 maskiner 5 minuter att göra 5 produkter. Hur lång tid skulle det ta 100 maskiner att göra 100 produkter?**   If it takes 5 machines 5 minutes to make 5 widgets, how long would it take 100 machines to make 100 widgets? |
| --- |

| ❑ | Antal minuter: ___________  Number of minutes |
| --- | --- |
| ❑ | Vet ej  Don’t know |

-------------------------------------Sidbrytning--------------------------------------------------------

| **6. En sjö är täckt av näckrosblad. Varje dag fördubblas täcket i storlek. Om det tar 48 dagar för näckrosorna att täcka hela sjön, hur lång tid tar det för täcket att täcka halva sjön?** In a lake, there is a patch of lily pads. Every day, the patch doubles in size. If it takes 48 days for the patch to cover the entire lake, how long would it take for the patch to cover half of the lake? |
| --- |

| ❑ | Antal dagar: ___________  Number of days |
| --- | --- |
| ❑ | Vet ej Don’t know |

-------------------------------------Sidbrytning--------------------------------------------------------

**Section 5**

| **I denna del undersöker vi vilka typer av citat som människor uppfattar som tänkvärda och meningsfulla. Nedan ser du ett antal citat. Vänligen läs varje citat och tänk på vad citatet betyder och vad det vill förmedla. Markera därefter på skalan i vilken grad du tycker att citatet förmedlar något som är tänkvärt och meningsfullt.**  **In this section we investigate what types of quotes that people perceive as meaningful and worthy of consideration. Below you see a number of quotes. Please read each quote and think about what the quote mean and what it wants to convey. Then mark on the scale to what extent you think the quote convey something that is meaningful and worth consideration** |
| --- |

|  | Inte det  minsta tänkvärt/  meningsfullt Not at all meaningful/worth considering | Knappast tänkvärt/ meningsfullt Hardly worth meaningful/worth considering | Lite  tänkvärt/ meningsfullt Slightly meaningful/worth considering | Ganska tänkvärt/ meningsfullt Rather meaningful/worth considering | Tänkvärt/ meningsfullt Meaningful/worth considering | Väldigt  tänkvärt/ meningsfullt Very meaningful/worth considering |
| --- | --- | --- | --- | --- | --- | --- |
| 1. En flod kan skära genom en sten, inte på grund av sin kraft, utan på grund av sin uthållighet  A river cuts through a rock, not because of its power but its persistence | ❑ | ❑ | ❑ | ❑ | ❑ | ❑ |
| 2. Den dolda meningen förvandlar den abstrakta skönheten The hidden meaning transforms the abstract beauty | ❑ | ❑ | ❑ | ❑ | ❑ | ❑ |
| 3. Framtiden förklarar irrationell fakta för den som söker The future elucidates irrational facts for the seeking person | ❑ | ❑ | ❑ | ❑ | ❑ | ❑ |
| 4. Du är inte bara ansvarig för det du säger, utan även för det du inte säger You are not only responsible for the things you say, but also for the things you do not say | ❑ | ❑ | ❑ | ❑ | ❑ | ❑ |
| 5. God hälsa och tolerans ger verkligheten kreativitet Health and tolerance provides creativity for the future | ❑ | ❑ | ❑ | ❑ | ❑ | ❑ |
| 6. Vi har andras fel inför ögonen men våra egna bakom vår rygg We have others flaws before our eyes, but our own flaws behind our back | ❑ | ❑ | ❑ | ❑ | ❑ | ❑ |
| 7. Din lärare kan öppna dörren, men du måste själv stiga in Your teacher can open the door, but you have to step in | ❑ | ❑ | ❑ | ❑ | ❑ | ❑ |
| 8. Din rörelse transformerar universella observationer Your movement transforms universal observations | ❑ | ❑ | ❑ | ❑ | ❑ | ❑ |
| 9. Den som aldrig gjort ett misstag har aldrig provat något nytt The person who never made a mistake never tried something new | ❑ | ❑ | ❑ | ❑ | ❑ | ❑ |
| 10. Helheten tystar oändliga fenomen The whole silence infinite phenomena | ❑ | ❑ | ❑ | ❑ | ❑ | ❑ |
| 11. Inbillade smärtor gör inte mindre ont för att de är inbillade Imagined pain does not hurt less because it is imagined | ❑ | ❑ | ❑ | ❑ | ❑ | ❑ |
| 12. Det osynliga är bortom all ny tidlöshet The invisible is beyond all new immutability | ❑ | ❑ | ❑ | ❑ | ❑ | ❑ |
| 13. Det oförklarliga berör universums inneboende erfarenheter  The unexplainable touches on the inherent experiences of the universe | ❑ | ❑ | ❑ | ❑ | ❑ | ❑ |
| 14. Det är en sak att bli frestad, men en helt annan att falla för frestelsen It is one thing to be tempted but quite another to fall for the temptation | ❑ | ❑ | ❑ | ❑ | ❑ | ❑ |

-------------------------------------Sidbrytning--------------------------------------------------------

**Section 6**

**All participants read all three dilemmas in randomized order. One third of the participants responded only to the blue should-questions, one third only on the purple should-questions and one third only on the orange moral choice questions (same question for all three dilemmas).**

|  |
| --- |

-------------------------------------Sidbrytning--------------------------------------------------------

| **I denna del kommer du att få läsa tre dilemman. Vänligen tänk dig att du jobbar på en organisation som bekämpar olika sjukdomar. I ditt jobb ingår det att besluta om vilka av de föreslagna hjälpprojekten som ska finansieras och vilka som inte ska finansieras. Det innebär att du måste välja vilket av två presenterade hjälpprojekt (Projekt A eller Projekt B) som ska finansieras. De båda projekten kostar lika mycket och du måste välja ett av projekten. Det finns inga regler eller direktiv om hur du ska välja utan du kan ta beslutet helt på egen hand. Du behöver inte rättfärdiga valet för någon.**  **Läs dilemmabeskrivningarna noga och svara genom att markera den siffra som bäst representerar ditt svar. Dilemmasituationerna kan tyckas hypotetiska och orealistiska men vänligen tänk dig att det som står i texten stämmer helt.**  **In this section you will read three dilemmas. Please imagine that you work for an organization preventing different diseases. Part of your job is to decide which suggested helping projects that should be financed and which that should not be financed. This means that you need to choose which of two presented helping projects (Project A or Project B) that will be financed. Both projects cost equally much and you need to choose one of the projects. There are no roles or directives for how you are supposed to choose, so you can take the decision all on your own. You do not need to justify your choice for anyone.**  **Read the dilemma descriptions carefully and answer by marking the number that best represent your answer. The dilemma situations might be seen as hypothetical and unrealistic but please imagine that the information in the text is accurate.**  **Dilemma 1**  **Projekt A beräknas kunna rädda livet på 25 barn i Sverige som lider av en allvarlig sjukdom**  **Projekt B beräknas kunna rädda livet på cirka 50 barn på den afrikanska landsbygden som lider av en annan lika allvarlig sjukdom. Du kan bara stödja ett av projekten.**  **Dina två valmöjligheter är:**  **(A) rädda livet på 25 barn i Sverige.**  **(B) rädda livet på 50 barn på den afrikanska landsbygden.**  **Project A is expected to save the life of 25 children in Sweden suffering from a severe disease**  **Project B is expected to save the life of 50 children living on the African countryside suffering from an equally severe disease. You can only support one of the projects.**  **Your two possible choices are:**   1. **Save the life of 25 children in Sweden** 2. **Save the life of 50 children on the African countryside**   **Dilemma 2**  **Projekt A beräknas kunna rädda livet på 25 barn som i detta nu lider av en specifik mycket allvarlig sjukdom.**  **Projekt B beräknas kunna rädda livet på cirka 50 framtida barn som kommer drabbas av en annan lika allvarlig sjukdom om cirka fem-tio år.**  **Dina två valmöjligheter är:**  **(A) rädda livet på 25 existerande barn som är sjuka nu.**  **(B) rädda livet på 50 framtida barn som ännu inte är födda.**  **Project A is expected to save the life of 25 existing children now suffering from a specific very severe disease**  **Project B is expected to save the life of 50 future children that will suffer from an equally severe disease 5-10 years from now. You can only support one of the projects.**  **Your two possible choices are:**  **(A) Save the life of 25 now existing children**  **(B) Save the life of 50 future children not yet born**  **Dilemma 3**  **Projekt A beräknas kunna rädda livet på 25 personer som lider av en sjukdom som framförallt tycks drabba människor som motionerar mycket.**  **Projekt B beräknas kunna rädda livet på 50 personer som lider av en annan allvarlig sjukdom som framförallt tycks drabba människor som äter onyttigt.**  **Dina två valmöjligheter är:**  **(A) rädda livet på 25 personer som lider av sjukdomen som drabbar de som motionerar.**  **(B) rädda livet på 50 personer som lider av sjukdomen som drabbar de som äter onyttigt.**  **Project A is expected to save the life of 25 persons suffering from a very severe disease that seems to happen only to to people that exercise a lot**  **Project B is expected to save the life of 50 persons suffering from an equally severe disease that seems to happen only to people eating unhealthy**  **Your two possible choices are:**  **(A) Save the life of 25 persons that do exercise**  **(B) Save the life of 50 persons that eat unhealthy**  **Vad skulle du göra?**  **What would you do?**  **Vad borde du göra?**  **What should you do?**  **Vad är det moraliska valet?**  **What is the moral choice?** |
| --- |

| (Markera hur pass övertygad du är över ditt val)  (Mark how convinced you are about your choice) |
| --- |

| Självklart skulle jag rädda livet på 25 barn i Sverige  Of course i would save 25 children in Sweden | Troligen skulle jag rädda livet på 25 barn i Sverige Probably i would save 25 children in Sweden | Troligen skulle jag rädda livet på 50 barn i Afrika Probably i would save 50 children in Africa | Självklart skulle jag rädda livet på 50 barn i Afrika Of course i would save 50 children in Africa |
| --- | --- | --- | --- |
| ❑ | ❑ | ❑ | ❑ |

| Självklart borde jag rädda livet på 25 barn i Sverige  Of course i should save 25 children in Sweden | Troligen borde jag rädda livet på 25 barn i Sverige Probably i should save 25 children in Sweden | Troligen borde jag rädda livet på 50 barn i Afrika Probably i should save 50 children in Africa | Självklart borde jag rädda livet på 50 barn i Afrika Of course i should save 50 children in Africa |
| --- | --- | --- | --- |
| ❑ | ❑ | ❑ | ❑ |

|  |  |  |  |  |
| --- | --- | --- | --- | --- |
| Självklart är det moraliska valet att rädda livet på 25 barn i Sverige  Of course the moral choice is to save 25 children in Sweden | | Troligen är det moraliska valet att rädda livet på 25 barn i Sverige Probably the moral choice is to save 25 children in Sweden | Troligen är det moraliska valet att rädda livet på 50 barn i Afrika Probably the moral choice is to save 50 children in Africa | Självklart är det moraliska valet att rädda livet på 50 barn i Afrika Of course the moral choice is to save 50 children in Africa |
| ❑ | | ❑ | ❑ | ❑ |

| Självklart skulle jag rädda livet på 25 existerande barn Of course i would save 25 existing children | Troligen skulle jag rädda livet på 25 existerande barn Probably i would save 25 existing children | Troligen skulle jag rädda livet på 50 framtida barn Probably I would save 50 future children | Självklart skulle jag rädda livet på 50 framtida barn Of course i would save 50 future children |
| --- | --- | --- | --- |
| ❑ | ❑ | ❑ | ❑ |

| Självklart borde jag rädda livet på 25 existerande barn Of course i should save 25 existing children | Troligen borde jag rädda livet på 25 existerande barn Probably i should save 25 existing children | Troligen borde jag rädda livet på 50 framtida barn Probably I should save 50 future children | Självklart borde jag rädda livet på 50 framtida barn Of course i should save 50 future children |
| --- | --- | --- | --- |
| ❑ | ❑ | ❑ | ❑ |

|  |  |  |  |  |
| --- | --- | --- | --- | --- |
| Självklart är det moraliska valet att rädda livet på 25 existerande barn  Of course the moral choice is to save 25 existing children | | Troligen är det moraliska valet att rädda livet på 25 existerande barn Probably the moral choice is to save 25 existing children | Troligen är det moraliska valet att rädda livet på 50 framtida barn Probably the moral choice is to save 50 future children | Självklart är det moraliska valet att rädda livet på 50 framtida barn Of course the moral choice is to save 50 future children |
| ❑ | | ❑ | ❑ | ❑ |

| Självklart skulle jag rädda livet på 25 personer med sjukdomen som drabbar de som motionerar  Of course I would save 25 persons with the disease that happen to those who exercise | Troligen skulle jag rädda livet på 25 personer med sjukdomen som drabbar de som motionerar Probably I would save 25 persons with the disease that happen to those who exercise | Troligen skulle jag rädda livet på 50 personer med sjukdomen som drabbar de som äter onyttigt  Probably I would save 50 persons with the disease that happen to those who eat unhealthy | Självklart skulle jag rädda livet på 50 personer med sjukdomen som drabbar de som äter onyttigt  Of course I would save 50 persons with the disease that happen to those who eat unhealthy |
| --- | --- | --- | --- |
| ❑ | ❑ | ❑ | ❑ |

| Självklart borde jag rädda livet på 25 personer med sjukdomen som drabbar de som motionerar  Of course I should save 25 persons with the disease that happen to those who exercise | Troligen borde jag rädda livet på 25 personer med sjukdomen som drabbar de som motionerar Probably I should save 25 persons with the disease that happen to those who exercise | Troligen borde jag rädda livet på 50 personer med sjukdomen som drabbar de som äter onyttigt  Probably I should save 50 persons with the disease that happen to those who eat unhealthy | Självklart borde jag rädda livet på 50 personer med sjukdomen som drabbar de som äter onyttigt  Of course I should save 50 persons with the disease that happen to those who eat unhealthy |
| --- | --- | --- | --- |
| ❑ | ❑ | ❑ | ❑ |

|  |  |  |  |  |
| --- | --- | --- | --- | --- |
| Självklart är det moraliska valet att rädda livet på 25 personer med sjukdomen som drabbar de som motionerar  Of course the moral choice is to save 25 persons with the disease that happen to those who exercise | | Troligen är det moraliska valet att rädda livet på 25 personer med sjukdomen som drabbar de som motionerar Probably the moral choice is to save 25 persons with the disease that happen to those who exercise | Troligen är det moraliska valet att rädda livet på 50 personer med sjukdomen som drabbar de som äter onyttigt  Probably the moral choice is to save 50 persons with the disease that happen to those who eat unhealthy | Självklart är det moraliska valet att rädda livet på 50 personer med sjukdomen som drabbar de som äter onyttigt  Of course the moral choice is to save 50 persons with the disease that happen to those who eat unhealthy |
| ❑ | | ❑ | ❑ | ❑ |

**Section 7**

-------------------------------------Sidbrytning--------------------------------------------------------

**Same scenarios as in Section 1- Which version you read in Section 1 determines which version you read here**

Version 1a in Section 1 = Version 2b here

Version 1b in Section 1 = Version 2a here

Version 2a in Section 1 = Version 1b here

Version 2b in Section 1 = Version 1a here

--------------------------------------------------------

**Section 8**

| **x1.**  **Hur glad känner du dig just nu?**  **How happy do you feel right now?** |
| --- |

|  | Inte alls  Glad  Not at all happy | Knappast  Glad  Hardly happy | Lite  Glad Slightly happy | Ganska  Glad Rather happy | Mycket  Glad  Very happy | Extremt  Glad  Extremely happy |
| --- | --- | --- | --- | --- | --- | --- |
|  | ❑ | ❑ | ❑ | ❑ | ❑ | ❑ |

-------------------------------------Sidbrytning--------------------------------------------------------

| **x2.**  **Hur ledsen känner du dig just nu?**  **How sad do you feel right now** |
| --- |

|  | Inte alls  Ledsen  Not at all sad | Knappast  Ledsen  Hardly sad | Lite  Ledsen  Slightly sad | Ganska  Ledsen  Rather sad | Mycket  Ledsen  Very sad | Extremt  Ledsen  Extremely sad |
| --- | --- | --- | --- | --- | --- | --- |
|  | ❑ | ❑ | ❑ | ❑ | ❑ | ❑ |

-------------------------------------Sidbrytning--------------------------------------------------------

| **x3.**  **Hur irriterad känner du dig just nu?**  **How irritated do you feel right now?** |
| --- |

|  | Inte alls  Irriterad  Not at all irritated | Knappast  Irriterad  Hardly irritated | Lite  Irriterad  Slightly irritated | Ganska  Irriterad  Rather irritated | Mycket  Irriterad  Very irritated | Extremt  Irriterad  Extremely irritatated |
| --- | --- | --- | --- | --- | --- | --- |
|  | ❑ | ❑ | ❑ | ❑ | ❑ | ❑ |

-------------------------------------Sidbrytning--------------------------------------------------------

**Section 9**

| **Nu är det bara några enstaka frågor kvar av undersökningen.**  **Om du nu markerar "Gå till avslutande frågor" så kommer du direkt till de avslutande frågorna.**  **Om du istället markerar "Fortsätt för Välgörenhet" så kommer du istället få svara på några extra frågor.**  **Om du väljer att fortsätta kommer dessutom vår forskargrupp att donera 5 kr till en valfri välgörenhetsorganisation (du kommer kunna välja vilken organisation som du vill stödja).**  **Det är helt upp till dig om du väljer att avsluta nu eller om du väljer att fortsätta för välgörenhet. Ditt val kommer inte kunna spåras till dig som person.**  **Vänligen välj ett av alternativen:**  **Only a few questions remain before you have completed this survey.**  **If you mark “Go to the concluding questions” now, you will be taken directly to the final questions.**  **If you instead mark “Continue for charity” you will instead be redirected and answer some additional questions. Also, if you choose to continue for charity, our research group will donate 5 SEK to a charitable organization of your choice (you will be able to pick which organization you want to donate to).**  **It is totally up to you if you choose to go directly to the end or if you want to continue for charity. Your choice will not be traceable to you as an individual.**  **Please choose one of the alternatives:** |
| --- |

| ❑ | Gå till avslutande frågor **-> to Section 10**  **Go to to concluding questions** |
| --- | --- |
| ❑ | Fortsätt för Välgörenhet **-> To questions below**  **Continue for charity** |

-------------------------------------Sidbrytning--------------------------------------------------------

| **TACK!**  **Här kommer ytterligare fyra frågor:**  **THANK YOU!**  **Here are four additional questions** |
| --- |

| Vänligen markera i vilken grad du håller med om varje påstående.  Please mark to what degree you agree with the following statements. |
| --- |

|  | Håller inte alls med  Strongly disagree | Håller till stor del inte med Moderatedly disagree | Håller delvis i nte med Slightly disagree | Neutral/  vet ej Neutral/do not know | Håller delvis med Slightly agree | Håller till stor del med Moderatedly agree | Håller med fullständigt Strongly agree |
| --- | --- | --- | --- | --- | --- | --- | --- |
| 1.Samhället fungerar bäst när det låter individer ta ansvar för sina egna liv utan att säga till dem vad de ska göra The society works the best when individuals are allowed to take responsibility for their own lives, without telling them what to do. | ❑ | ❑ | ❑ | ❑ | ❑ | ❑ | ❑ |
| 2. Staten borde göra mer för att främja samhällets mål, även om det innebär att begränsa individers frihet och valmöjligheter The government should do more to reach the goals of the society at large, even if it limits individual’s freedom and ability to choose | ❑ | ❑ | ❑ | ❑ | ❑ | ❑ | ❑ |
| 3. Staten borde sluta säga till människor hur de ska leva sina liv The government should stop telling people how to live their lives. | ❑ | ❑ | ❑ | ❑ | ❑ | ❑ | ❑ |
| 4. Staten borde begränsa människors val så att de inte kommer i vägen för vad som är bra för samhället  The government should limit people’s freedom of choices to avoid decisions that are not good for the society | ❑ | ❑ | ❑ | ❑ | ❑ | ❑ | ❑ |

-------------------------------------Sidbrytning--------------------------------------------------------

| **Markera den organisation som du vill att dina 5kr ska doneras till.**  **Mark the organization you want to donate your 5 SEK to.** |
| --- |

| ❑ | Unicef Sverige |
| --- | --- |
| ❑ | Svenska Röda Korset |
| ❑ | Läkare utan Gränser |
| ❑ | Rädda Barnen |
| ❑ | SOS Barnbyar |
| ❑ | Världsnaturfonden WWF |
| ❑ | Svenska Kyrkan |
| ❑ | Hjärt-Lungfonden |
| ❑ | Diakonia |
| ❑ | Fryshuset |
| ❑ | Barnfonden |
| ❑ | Reumatikerförbundet |
| ❑ | Cancerfonden |
| ❑ | Sverige för UNHCR |
| ❑ | Frälsningsarmén |
| ❑ | Barncancerfonden |
| ❑ | Stadsmissionen |
| ❑ | Hjärnfonden |
| ❑ | BRIS |
| ❑ | Plan Sverige |
| ❑ | We Effect |
| ❑ | Friends |
| ❑ | ActionAid |
| ❑ | Naturskyddsföreningen |
| ❑ | Min Stora Dag |
| ❑ | Amnesty International |
| ❑ | Greenpeace |
| ❑ | Sjöräddningssällskapet |
| ❑ | Erikshjälpen |
| ❑ | Hoppets Stjärna |
| ❑ | Hörselskadades Riksförbund |
| ❑ | Djurens Rätt |
| ❑ | Vi-Skogen |
| ❑ | Alzheimersfonden |

-------------------------------------Sidbrytning--------------------------------------------------------

**Section 10**

| **x1.**  **Hur glad känner du dig just nu?**  **How happy do you feel right now?** |
| --- |

|  | Inte alls  Glad  Not at all happy | Knappast  Glad  Hardly happy | Lite  Glad Slightly happy | Ganska  Glad Rather happy | Mycket  Glad  Very happy | Extremt  Glad  Extremely happy |
| --- | --- | --- | --- | --- | --- | --- |
|  | ❑ | ❑ | ❑ | ❑ | ❑ | ❑ |

-------------------------------------Sidbrytning--------------------------------------------------------

| **x2.**  **Hur ledsen känner du dig just nu?**  **How sad do you feel right now** |
| --- |

|  | Inte alls  Ledsen  Not at all sad | Knappast  Ledsen  Hardly sad | Lite  Ledsen  Slightly sad | Ganska  Ledsen  Rather sad | Mycket  Ledsen  Very sad | Extremt  Ledsen  Extremely sad |
| --- | --- | --- | --- | --- | --- | --- |
|  | ❑ | ❑ | ❑ | ❑ | ❑ | ❑ |

-------------------------------------Sidbrytning--------------------------------------------------------

| **x3.**  **Hur irriterad känner du dig just nu?**  **How irritated do you feel right now?** |
| --- |

|  | Inte alls  Irriterad  Not at all irritated | Knappast  Irriterad  Hardly irritated | Lite  Irriterad  Slightly irritated | Ganska  Irriterad  Rather irritated | Mycket  Irriterad  Very irritated | Extremt  Irriterad  Extremely irritatated |
| --- | --- | --- | --- | --- | --- | --- |
|  | ❑ | ❑ | ❑ | ❑ | ❑ | ❑ |

-------------------------------------Sidbrytning--------------------------------------------------------

**Section 11**

| **I denna sista del ska du svara på frågor om dig själv.**  **In this final section you are responding to questions about yourself.**  **Vad är ditt kön?**  **What is your sex/gender?** | |  |
| --- | --- | --- |
| (markera det du är) | | |
| ❑ | | Kvinna  Female |
| ❑ | | Man  Male |

-------------------------------------Sidbrytning--------------------------------------------------------

| **Vad är din nuvarande ålder?**  **What is your current age?** |  |
| --- | --- |
| (Skriv din ålder i rutan) | |

|  | _________________________ |
| --- | --- |

-------------------------------------Sidbrytning--------------------------------------------------------

| **Vilken är din högsta slutförda utbildning?**  **What is your highest completed education?** |  |
| --- | --- |
| (markera det som stämmer) | |

| ❑ | Ej slutfört grundskolan  Not finished junior high school |
| --- | --- |
| ❑ | Slutfört grundskola  Finished junior high school |
| ❑ | Slutfört gymnasiet  Finished high school |
| ❑ | Påbörjade studier vid högskola/universitet  Began studies at university/college |
| ❑ | Tagit examen på högskola/universitet  Graduated university/college |

-------------------------------------Sidbrytning--------------------------------------------------------

| **Hur ofta du läser religiösa texter, går i kyrkan, eller ber till Gud?**  **How often do you read religious texts, go to church or pray to God?** |  |
| --- | --- |
| (markera det alternativ som stämmer in bäst) | |

| ❑ | Aldrig  Never |
| --- | --- |
| ❑ | En gång per år  Once each year |
| ❑ | Några gånger per år  A few times each year |
| ❑ | En gång per månad  Once each month |
| ❑ | En gång per vecka  A few times a week |
| ❑ | Flera gånger i veckan  Several times a week |
| ❑ | Varje dag  Every day |

-------------------------------------Sidbrytning--------------------------------------------------------

| **Har du under det senaste året donerat pengar till välgörenhetsorganisationer eller andra ideella organisationer?**  **Have you at anytime the past year donated money to charity organizations or other non-profit organizations?** |
| --- |

| ❑ | Nej  No |
| --- | --- |
| ❑ | Ja  Yes |

-------------------------------------Sidbrytning--------------------------------------------------------

**Question to those answering yes on the previous question**

| **Ungefär hur mycket pengar donerade du totalt till välgörenhetsorganisationer eller andra ideella organisationer under det senaste året?**  **About how much did you donate in total to charity or other non-profit organizations during the past year** |
| --- |

| (skriv ett belopp i rutan) (write an amount) |
| --- |

| ______________________________ |
| --- |

-------------------------------------Sidbrytning--------------------------------------------------------

| **Olika välgörenhetsorganisationer har olika verksamhetsområden. Nedan är en lista med vanliga verksamhetsområden. Olika verksamhetsområden ligger olika människor varmast om hjärtat. Vänligen markera hur viktiga följande verksamhetsområden är för dig personligen.**  **Different charity organizations focus on different causes. Below is a list with common causes for charity organizations. Different charitable causes lie the closest to the heart of different people. Please mark how important the following charitable causes are for you personally.** |
| --- |

|  | Inte alls viktigt för mig  Not at all important for me | Inte särskilt viktigt för mig  Not so important for me | Lite viktigt för mig Slightly important for me | Viktigt för mig Important for me | Väldigt viktigt för mig Very important for me |
| --- | --- | --- | --- | --- | --- |
| Vård och behandling av sjuka barn och vuxna i Sverige  Treatment and care for sick children and adults in Sweden | ❑ | ❑ | ❑ | ❑ | ❑ |
| Vård och omsorg av äldre i Sverige  Treatment and care of elderly in Sweden | ❑ | ❑ | ❑ | ❑ | ❑ |
| Forskning om allvarliga sjukdomar som drabbar många svenskar (t.ex. olika Cancersjukdomar och Alzheimers)  Research about severe diseases that befall many Swedes (i.e. Alzheimers disease and Cancer) | ❑ | ❑ | ❑ | ❑ | ❑ |
| Socialt arbete och fattigdomsbekämpning i Sverige  Social work and poverty prevention in Sweden | ❑ | ❑ | ❑ | ❑ | ❑ |
| Hjälp och stöd till flyktingar som befinner sig i Sverige  Help and support for refugees currently in Sweden | ❑ | ❑ | ❑ | ❑ | ❑ |
| Politiskt arbete i Sverige  Political work in Sweden | ❑ | ❑ | ❑ | ❑ | ❑ |
| Vård och behandling av sjuka barn och vuxna i andra länder  Treatment and care of sick children and adults in other countries. | ❑ | ❑ | ❑ | ❑ | ❑ |
| Akutinsatser efter katastrofer i andra länder  Responses to disasters and acute situations in other countries. | ❑ | ❑ | ❑ | ❑ | ❑ |
| Forskning om allvarliga sjukdomar som drabbar många människor i andra länder (t.ex. Malaria och Infektionssjukdomar)  Research about severe diseases that befall many people in other countries (i.e. Malaria and infectious diseases) | ❑ | ❑ | ❑ | ❑ | ❑ |
| Socialt arbete och fattigdomsbekämpning i andra länder  Social work and poverty prevention in other countries | ❑ | ❑ | ❑ | ❑ | ❑ |
| Hjälp och stöd till flyktingar som befinner sig i andra länder  Help and support for refugees currently in other countries | ❑ | ❑ | ❑ | ❑ | ❑ |
| Politiskt arbete i andra länder  Political work in other countries | ❑ | ❑ | ❑ | ❑ | ❑ |
| Naturskydd och hållbar utveckling  Environmental work and sustainable development | ❑ | ❑ | ❑ | ❑ | ❑ |
| Djurs rättigheter och välmående  Animal rights and animal well being | ❑ | ❑ | ❑ | ❑ | ❑ |
| Religiöst arbete  Religious work | ❑ | ❑ | ❑ | ❑ | ❑ |
| Konst och kultur  Fine arts and culture | ❑ | ❑ | ❑ | ❑ | ❑ |
| Främjandet av idrott  Sports | ❑ | ❑ | ❑ | ❑ | ❑ |

-------------------------------------Sidbrytning--------------------------------------------------------

| **Har du under det senaste året gett pengar eller på annat sätt hjälpt tiggande EU-migranter?**  **Have you anytime during the past year given money or in other ways helped begging EU-migrants?** |  |
| --- | --- |
| (markera det som stämmer) | |

| ❑ | Nej, aldrig  No, never |
| --- | --- |
| ❑ | Ja, någon enstaka gång  Yes, once |
| ❑ | Ja, några gånger  Yes, a few times |
| ❑ | Ja, många gånger  Yes, many times |
| ❑ | Ja, varje vecka  Yes, every week |

-------------------------------------Sidbrytning--------------------------------------------------------

| **Tycker du att det ska vara lagligt att tigga i Sverige?**  **Do you think it should be legal to beg in Sweden?** |  |
| --- | --- |
| (markera det som stämmer) | |

| ❑ | Nej, absolut inte  No, absolutely not |
| --- | --- |
| ❑ | Nej, troligtvis inte  No, probably not |
| ❑ | Tveksam  Not sure |
| ❑ | Ja, troligtvis  Yes, probably |
| ❑ | Ja, absolut  Yes, absolutely |

-------------------------------------Sidbrytning--------------------------------------------------------

| **Uppfattar du dig själv som politiskt till vänster eller till höger på den politiska skalan?**  **Do your perceive yourself as being to the left or to the right on the political scale?** |  |
| --- | --- |
| (markera det som stämmer) | |

| ❑ | Väldigt långt till vänster  Very far to the let |
| --- | --- |
| ❑ | Långt till vänster  Far to the left |
| ❑ | Ganska mycket till vänster  Pretty much to the left |
| ❑ | Svag tendens till vänster  Weakly to the left |
| ❑ | Mitten/ Varken vänster eller höger  The middle/ neither left nor right |
| ❑ | Svag tendens till höger  Weakly to the right |
| ❑ | Ganska mycket till höger  Pretty much to the right |
| ❑ | Långt till höger  Far to the right |
| ❑ | Väldigt långt till höger  Very far to the right |

-------------------------------------Sidbrytning--------------------------------------------------------

| **Om det var val idag, vilket parti skulle du rösta på?**  **If it was an election today, which party would you vote for?** |
| --- |

| ❑ | Socialdemokraterna  Social democratic party |
| --- | --- |
| ❑ | Moderaterna  The Moderates |
| ❑ | Sverigedemokraterna  The Sweden democrates |
| ❑ | Miljöpartiet  The Green Party |
| ❑ | Centerpartiet  The Center party |
| ❑ | Vänsterpartiet  The Left party |
| ❑ | Liberalerna (f.d. Folkpartiet)  The Liberal party |
| ❑ | Kristdemokraterna  The Christian Democratic party |
| ❑ | Annat (skriv i rutan): ___________  Other (write which) |
| ❑ | Vill inte uppge/Vet inte  Do not want to say/do not know |
