# Supplementary material for: Bullshit-sensitivity predicts prosocial behavior
Source: PLoS One. 2018 Jul 31;13(7):e0201474. doi: 10.1371/journal.pone.0201474 (PMC6067753; doi:10.1371/journal.pone.0201474)
Supplement: S3 File — (DOCX) [file pone.0201474.s003.docx]

**Supplementary Material 3.**

**Key for understanding variables in dataset: (datafile “[S4]Raw data_BS”). Variables in bold are the ones included in the manuscript.**

| **Volunteering_decision_0NO_1YES** | Volunteering decision:  (0 = Did not volunteer for charity, 1 = Did volunteer for charity) |
| --- | --- |
| **Donation_experience_0NO_1YES** | Donation experience:  (0 = Did not donate to charity during the past year, 1 = Did donate to charity during the past year) |
| **Meaningfulness_on_bullshit_sentences** | Bullshit receptivity: Participant’s mean perceived meaningfulness for the bullshit sentences |
| **Meaningfulness_on_genuine_sentences** | Profoundness receptivity: Participant’s mean perceived meaningfulness for the genuinely profound sentences |
| **Bullshit_sensitivity** | Bullshit sensitivity:  (Calculated by taking profoundness receptivity minus bullshit reciptivity) |
| **Sex_0female_1male** | Participants sex:  (0 = female, 1 = male) |
| **Current_age** | Participants age: |
| **Education** | Participants highest level of education:  (1 = not completed elementary school, 2 = completed elementary school, 3 = completed high school, 4 = studied on university/collage, 5 = graduated university/collage) |
| **Religiosity_4items** | Participants level of religiosity: (R1, R2, R3, R4) (calculated from four items, α = .80) |
| **Political_self_placement** | Participants political self-placement:  (1 = very far to the left, 2 = far to the left, 3 = mostly to the left, 4 weak leaning to the left, 5 = neither left nor right/the middle, 6 weak leaning to the right, 7 = mostly to the right, 8 = far to the right, 9 = very far to the right) |
| **Numeracy_correct_responses** | Participant’s numeracy score  (number of correct responses on the three numeracy-questions) |
| **CRT_correct_responses** | Participant’s cognitive reflection ability score  (number of correct responses on the three CRT-questions) |
| **Cog_ability** | Numeracy score plus cognitive reflection ability score (theoretical score 0-6 correct responses) |
| **Time_max60** | Participant’s time spent [in minutes] for completing the survey  (participants who used more than an hour were adjusted to receive a score of 60) |
|  |  |
|  |  |
| Time | Participant’s time spent [in minutes] for completing the survey  (not adjusted) |
| T1_MotCog_scenario | Section 1. Motivated cognition - Which scenario did the participant read: 1 = 1a-neutral scenario, increase is correct answer, 2 = 1b-neutral scenario, decrease is correct answer, 3 = 2a-loaded scenario, increase is correct, 4 = 2b-loaded scenario, decrease is correct. |
| T1_MotCog_correct | Section 1. Motivated cognition – Did the participant respond correctly to the question (0 = incorrect, 1 = correct) |
| Ideology1_ecoequality_vs_ecofreedom | Participants ideological self-placement 1 [economic equality vs. economic freedom]: (1 = economic equality much more important, 2 = economic equality more important, 3 = economic equality slightly more important, 4 = both equally important, 5 = economic freedom slightly more important, 6 = economic freedom more important, 7 = economic freedom much more important) |
| Ideology2R_liberalism_vs_conservatism | Participants ideological self-placement 2 [liberalism vs. conservatism in social questions]: (1 = Strongly Liberal, 2 = Liberal, 3 = Slightly Liberal, 4 = Neither Liberal nor Conservative / the middle, 5 = Slightly Conservative, 6 = Conservative, 7 = Strongly Conservative). This variable was responded to inversely in the questionnaire but have been reversed to ease interpretation. |
| Ideology3_worldcitizen_vs_swede | Participants ideological self-placement 3 [identifying as a world-citizen vs. as a Swede]: (1 = Much more as a world-citizen, 2 = More as a world-citizen, 3 = Slightly more as a world-citizen, 4 = Equally much/little as a world-citizen and as a Swede, 5 = Slightly more as a Swede, 6 = More as a Swede, 7 = Much more as a Swede) |
| Ideology4R_othersfeelings_vs_freespeech | Participants ideological self-placement 4 [importance of protecting other’s feelings vs. importance of protecting the free speech ]: (1 = Protecting others’ feeling much more important, 2 = Protecting others’ feeling more important, 3 = Protecting others’ feeling slightly more important, 4 Equally important /unimportant 5 = Protecting free speech slightly more important, 6 = Protecting free speech more important, 7 = Protecting free speech much more important) This variable was responded to inversely in the questionnaire but have been reversed to ease interpretation. |
| Ideology2_conservatism_vs_liberalism | Original (not reversed) version of the question about participants ideological self-placement 2 [liberalism vs. conservatism in social questions] (1 = Strongly Conservative, 2 = Conservative, 3 = Slightly Conservative, 4 = Neither Conservative nor Liberal / the middle, 5 = Slightly Liberal, 6 = Liberal, 7 = Strongly Liberal). |
| Ideology4_freespeech_vs_othersfeelings | Original (not reversed) version of the question about participants ideological self-placement 4 [importance of protecting other’s feelings vs. importance of protecting the free speech ] (1 = Protecting free speech much more important, 2 = Protecting free speech more important, 3 = Protecting free speech slightly more important, 4 Equally important /unimportant 5 = Protecting others’ feeling slightly more important, 6 = Protecting others’ feeling more important, 7 = Protecting others’ feeling much more important) |
| Religiosity1 | Religious belief [R1]. I feel that on a higher level all of us share a common bond [Jag känner att vi alla har ett gemensamt band på ett högre plan.]  (1 = strongly disagree, 7 = strongly agree) |
| Spirituality1 | Spiritul universalism: All life is interconnected [Allt liv är sammanvävt.] |
| Religiosity2 | Religious belief [R2]. There is a higher plane of consciousness or spirituality that binds all people [Det finns en högre nivå av medvetande och andlighet som binder samman alla människor.]  (1 = strongly disagree, 7 = strongly agree) |
| Spirituality2 | Spiritual universalism: Although individual people may be difficult, I feel an emotional bond with all of humanity [Även om enskilda personer kan vara besvärliga känner jag ett känslomässigt band med hela mänskligheten.]  (1 = strongly disagree, 7 = strongly agree) |
| Religiosity3 | Religious identity [R3]. Religion is an important part of my life [Religion är en viktig del av mitt liv]  (1 = strongly disagree, 7 = strongly agree) |
| Religious_activities | Religious activities [R4]. How often do you read religious texts, go to church or pray to God? [Hur ofta du läser religiösa texter, går i kyrkan, eller ber till Gud?] (1 = never, 7 = every day) |
| Numeracy1 | Numeracy question 1. "In an American lottery, the chance of winning a $10 prize is 1%. What is your best guess  about how many people would win a $10 prize if  1000 people each buy a single ticket to the lottery?” |
| Numeracy1_response | Response on numeracy question 1 |
| Numeracy1_correct | Was the participant’s response on numeracy question 1 correct [10 is correct] ( 0 = incorrect, 1 = correct) |
| Numeracy2 | Numeracy question 2. ”Imagine we are throwing a five-sided die 50 times. On average, out of these 50 throws how many times would this five-sided die show an odd number (1, 3  or 5)?” |
| Numeracy2_response | Response on numeracy question 2 |
| Numeracy2_correct | Was the participant’s response on numeracy question 2 correct [30 is correct] ( 0 = incorrect, 1 = correct) |
| Numeracy3 | Numeracy question 3. “Out of 1,000 people in a small town 500 are members of a choir. Out of these 500 members in the  choir 100 are men. Out of the 500 inhabitants that  are not in the choir 300 are men. What is the probability  that a randomly drawn man is a member of the  choir? (please indicate the probability in percent).” |
| Numeracy3_response | Response on numeracy question 3 |
| Numeracy3_correct | Was the participant’s response on numeracy question 3 correct [25 is correct] ( 0 = incorrect, 1 = correct) |
| CRT1 | CRT question 1. A bat and a ball cost $110 in total. The bat costs $100 more than the ball. How much does the ball cost? |
| CRT1_response | Response on CRT question 1 |
| CRT1_correct | Was the participant’s response on CRT question 1 correct [5 is correct] ( 0 = incorrect, 1 = correct) |
| CRT2 | CRT question 2. “If it takes 5 machines 5 minutes to make 5 widgets, how long would it take 100 machines to make 100 widgets? _____ minutes” |
| CRT2_response | Response on CRT question 2 |
| CRT2_correct | Was the participant’s response on CRT question 2 correct [5 is correct] ( 0 = incorrect, 1 = correct) |
| CRT3 | CRT question 3. “In a lake, there is a patch of lily pads. Every day, the patch doubles in size. If it takes 48 days for the patch to cover the entire lake, how long would it take for the patch to cover half of the lake? _____ days” |
| CRT3_response | Response on CRT question 3 |
| CRT3_correct | Was the participant’s response on CRT question 3 correct [47 is correct] ( 0 = incorrect, 1 = correct) |
| BS1_Genuine | A river cuts through a rock, not because of its power but its persistence  En flod kan skära genom en sten, inte på grund av sin kraft, utan på grund av sin uthållighet |
| BS2_Bullshit | The hidden meaning transforms the abstract beauty  Den dolda meningen förvandlar den abstrakta skönheten |
| BS3_Bullshit | The future elucidates irrational facts for the seeking person Framtiden förklarar irrationell fakta för den som söker |
| BS4_Genuine | You are not only responsible for the things you say, but also for the things you do not say  Du är inte bara ansvarig för det du säger, utan även för det du inte säger |
| BS5_Bullshit | Health and tolerance provides creativity for the future  God hälsa och tolerans ger verkligheten kreativitet |
| BS6_Genuine | We have others flaws before our eyes, but our own flaws behind our back  Vi har andras fel inför ögonen men våra egna bakom vår rygg |
| BS7_Genuine | Your teacher can open the door, but you have to step in  Din lärare kan öppna dörren, men du måste själv stiga in |
| BS8_Bullshit | Your movement transforms universal observations  Din rörelse transformerar universella observationer |
| BS9_Genuine | The person who never made a mistake never tried something new  Den som aldrig gjort ett misstag har aldrig provat något nytt |
| BS10_Bullshit | The whole silence infinite phenomena  Helheten tystar oändliga fenomen |
| BS11_ Genuine | Imagined pain does not hurt less because it is imagined Inbillade smärtor gör inte mindre ont för att de är inbillade |
| BS12_Bullshit | The invisible is beyond all new immutability  Det osynliga är bortom all ny tidlöshet |
| BS13_Bullshit | The unexplainable touches on the inherent experiences of the universe  Det oförklarliga berör universums inneboende erfarenheter |
| BS14_Genuine | It is one thing to be tempted but quite another to fall for the temptation  Det är en sak att bli frestad, men en helt annan att falla för frestelsen |
| Meaningfulness_proneness | Meaningfulness proneness:  (Calculated by taking the mean perceived meaningfulness of genuinely profound sentences plus mean perceived meaningfulness of bullshit sentences |
| Dilemma_condition | Did participant respond to the dilemmas in the “would you”, “should you” or “moral choice” frame: (1 = would you, 2 = should you, 3 = moral choice) |
| Dilemma1_response | Ingroup-dilemma  (1 = certainly save 25 Swedish children  2 = probably save 25 Swedish children  3 = probably save 50 African children  4 = probably save 50 African children) |
| Dilemma2_response | Temporal distance-dilemma  (1 = certainly save 25 now existing children  2 = probably save 25 now existing children  3 = probably save 50 future children  4 = probably save 50 future children) |
| Dilemma3_response | Innocentness-dilemma  (1 = certainly save 25 people living a healthy life  2 = probably save 25 people living a healthy life  3 = probably save 50 people living an unhealthy life  4 = probably save 50 people living an unhealthy life) |
| T2_MotCog_scenario | Section 7. Motivated cognition - Which scenario did the participant read: 1 = 1a-neutral scenario, increase is correct answer, 2 = 1b-neutral scenario, decrease is correct answer, 3 = 2a-loaded scenario, increase is correct, 4 = 2b-loaded scenario, decrease is correct. |
| T2_MotCog_correct | Section 7. Motivated cognition – Did the participant respond correctly to the question (0 = incorrect, 1 = correct) |
| Time1_happy | Section 8: How happy do you feel right now  (1 = not at all happy, 6 = extremely happy) |
| Time1_sad | Section 8: How sad do you feel right now  (1 = not at all sad, 6 = extremely sad) |
| Time1_irritated | Section 8: How irritated do you feel right now  (1 = not at all irritated, 6 = extremely irritated) |
| Choice_of_organization | Which organization did the participant choose to donate money to?  (from a list of 30 of the most famous organizations in Sweden) |
| Time2_happy | Section 10: How happy do you feel right now  (1 = not at all happy, 6 = extremely happy) |
| Time2_sad | Section 10: How sad do you feel right now  (1 = not at all sad, 6 = extremely sad) |
| Time2_irritated | Section 10: How irritated do you feel right now  (1 = not at all irritated, 6 = extremely irritated) |
| Donation_amount_numeric | [Only given to those answering yes on the donation experience question] Around how much did you donate to charity organizations and other non-profit organizations during the past year? (Those answering above 10000 SEK adjusted down to 10001) |
| Omrade1_sjuka_i_sverige | How important do you think this charitable cause is? [Sick people in Sweden] (1 = not at all important, 5 = highly important to me) |
| Omrade2_äldre_i_sverige | How important do you think this charitable cause is? [Elderly in Sweden] (1 = not at all important, 5 = highly important to me) |
| Omrade3_forskning_till_sverige | How important do you think this charitable cause is? [Research benefitting people in Sweden] (1 = not at all important, 5 = highly important to me) |
| Omrade4_fattigdom_i_sverige | How important do you think this charitable cause is? [Preventing poverty in Sweden] (1 = not at all important, 5 = highly important to me) |
| Omrade5_flyktingar_i_sverige | How important do you think this charitable cause is? [Refugees in Sweden] (1 = not at all important, 5 = highly important to me) |
| Omrade6_politiskt_i_sverige | How important do you think this charitable cause is? [Political work in Sweden] (1 = not at all important, 5 = highly important to me) |
| Omrade7_sjuka_utomlands | How important do you think this charitable cause is? [Sick people abroad] (1 = not at all important, 5 = highly important to me) |
| Omrade8_akutinsatser_utomlands | How important do you think this charitable cause is? [Emergencies abroad] (1 = not at all important, 5 = highly important to me) |
| Omrade9_forskning_utomlands | How important do you think this charitable cause is? [Research benefitting non Swedish people] (1 = not at all important, 5 = highly important to me) |
| Omrade10_fattigdom_utomlands | How important do you think this charitable cause is? [Poverty abroad] (1 = not at all important, 5 = highly important to me) |
| Omrade11_flyktingar_utomlands | How important do you think this charitable cause is? [Refugees abroad] (1 = not at all important, 5 = highly important to me) |
| Omrade12_politiskt_utomlands | How important do you think this charitable cause is? [Political work abroad] (1 = not at all important, 5 = highly important to me) |
| Omrade13_naturskydd | How important do you think this charitable cause is? [Protecting the environment] (1 = not at all important, 5 = highly important to me) |
| Omrade14_djur | How important do you think this charitable cause is? [Animal welfare] (1 = not at all important, 5 = highly important to me) |
| Omrade15_religiöst | How important do you think this charitable cause is? [Religious work] (1 = not at all important, 5 = highly important to me) |
| Omrade16_konstkultur | How important do you think this charitable cause is? [Art and culture] (1 = not at all important, 5 = highly important to me) |
| Omrade17_idrott | How important do you think this charitable cause is? [Sports] (1 = not at all important, 5 = highly important to me) |
| Begging_giving | Have you during the past year given money or in any other way helped begging EU-migrants (1 = no never, 5 = yes every week) |
| Begging_legal | Do you think it should be legal to beg in Sweden? (1 = no absolutely not, 5 = yes absolutely) |
| Political_party_preference | If there was an election today, which party would you vote for?  1 = Social democratic party, 2 = moderate party, 3 Swedish democratic party, 4 = green party, 5 = center party, 6 = left party, 7 = liberal party, 8 = Christian democratic party, 9 = other party, 10 = don’t know / do not want to say. |
|  |  |
|  | *All items below (1 = strongly disagree, 7 = strongly agree)* |
| Q11_1 | Compassion for those who are suffering is the most crucial virtue. |
| Q11_2 | When the government makes laws, the number one principle should be ensuring that everyone is treated fairly. |
| Q11_3 | I am proud of my country’s history. |
| Q11_4 | Respect for authority is something all children need to learn. |
| Q11_5 | People should not do things that are disgusting, even if no one is harmed. |
| Q11_6 | One of the worst things a person could do is hurt a defenseless animal. |
| Q11_7 | Justice is the most important requirement for a society. |
| Q11_8 | People should be loyal to their family members, even when they have done something wrong. |
| Q11_9 | Men and women each have different roles to play in society. |
| Q11_10 | I would call some acts wrong on the grounds that they are unnatural. |
| Q11_11 | It can never be right to kill a human being. |
| Q11_12 | I think it’s morally wrong that rich children inherit a lot of money while poor children inherit nothing. |
| Q11_13 | It is more important to be a team player than to express oneself. |
| Q11_14 | If I were a soldier and disagreed with my commanding officer’s orders, I would obey anyway because that is my duty. |
| Q11_15 | Chastity is an important and valuable virtue. |
| Q12_1 | Misfortune is least likely to strike worthy decent people. |
| Q12_2 | Generally, people deserve what they get in this world. |
| Q12_3 | People will experience good fortune if they themselves are good. |
| Q12_4 | By and large, good people get what they deserve in this world. |
| Q12_5 | People’s misfortunes result from mistakes they have made. |
| Q12_6 | Through our actions we can prevent bad things from happening to us. |
| Q12_7 | If people took preventive actions, most misfortune could be avoided. |
| Q12_8 | When bad things happen, it is typically because people have not taken the necessary actions to protect themselves. |
| Q14_1 | If people were treated more equally in this country we would have many fewer problems |
| Q14_2 | It is a mistake to try to guarantee an equal distribution of resources between rich and poor |
| Q14_3 | Our society should do whatever is necessary to make sure that everyone has equal opportunities to succeed |
| Q14_4 | Substantial income differences are needed to motivate people to work hard enough to improve the economy |
| Q14_5 | This country would be better off if there were more emphasis on traditional family ties. |
| Q14_6 | Our customs and national heritage are the things that have made us great, and certain people should be made to show greater respect for them. |
| Q14_7 | If you start changing things very much, you often end up making them worse. |
| Q14_8 | Changing any institution (e.g., government, religion, business) is risky, so it is better to change at a slow than a rapid pace. |
| Q40_1 | The society works the best when individuals are allowed to take responsibility for their own lives, without telling them what to do. |
| Q40_2 | The government should do more to reach the goals of the society at large, even if it limits individual’s freedom and ability to choose |
| Q40_3 | The government should stop telling people how to live their lives. |
| Q40_4 | The government should limit people’s freedom of choices to avoid decisions that are not good for the society |
|  |  |
|  |  |
|  | *All items below (1 = not at all relevant, 7 = extremely relevant)* |
| Q15_1 | Whether or not someone suffered emotionally |
| Q15_2 | Whether or not some people were treated differently than others |
| Q15_3 | Whether or not someone’s action showed love for his or her country |
| Q15_4 | Whether or not someone showed a lack of respect for authority |
| Q15_5 | Whether or not someone violated standards of purity and decency |
| Q15_6 | Whether or not someone cared for someone weak or vulnerable |
| Q15_7 | Whether or not someone acted unfairly |
| Q15_8 | Whether or not someone did something to betray his or her group |
| Q15_9 | Whether or not someone conformed to the traditions of society |
| Q15_10 | Whether or not someone did something disgusting |
| Q15_11 | Whether or not someone was cruel |
| Q15_12 | Whether or not someone was denied his or her rights |
| Q15_13 | Whether or not someone showed a lack of loyalty |
| Q15_14 | Whether or not an action caused chaos or disorder |
| Q15_15 | Whether or not someone acted in a way that God would approve of |
|  |  |
