# Supplementary material for: Bullshit-sensitivity predicts prosocial behavior
Source: PLoS One. 2018 Jul 31;13(7):e0201474. doi: 10.1371/journal.pone.0201474 (PMC6067753; doi:10.1371/journal.pone.0201474)
Supplement: S5 File — Appendix A: Structural Equation Modelling. (DOCX) [file pone.0201474.s005.docx]

**Appendix A: Structural Equation Modeling (SEM)**

To complement the regression analyses, we also analyzed the data with SEM to control for the influence of covariates while accounting for measurement error. The reason we did this is that traditional regression models used for this purpose lead to high Type 1 error rates when reliability is imperfect, and particularly so when sample size is large [40]. We ran SEM in AMOS 24.0, basing calculations on the covariance matrix and the maximum likelihood method. We evaluated model fit in terms of the χ^2^ Goodness-of-fit test; the Comparative Fit Index (CFI), which estimates goodness of fit compared to the independence model; the Root Mean Square Error of Approximation (RMSEA), which estimates parsimony-adjusted lack of fit in relation to a perfect model, with 90% confidence intervals; and the Akaike Information Criterion (AIC), which estimates loss of information when a model is made simpler. Widely employed rules of thumb suggest that CFI estimates around .95 and RMSEA estimates below .06 indicate acceptable model fit [41]. However, evaluating CFI this way is meaningful only if the RMSEA of the independence model is above .158 [42]. We report standardized path coefficients for the paths from bullshit and profoundness receptivity to the donation variables with bias-corrected 95% bootstrap confidence intervals generated through 10000 resamples.

In our models, bullshit receptivity and profoundness receptivity were represented by latent factors, each with seven individual items as indicators (*λ* ≥ .60 for bullshit receptivity and *λ* ≥ .50 for profoundness receptivity). We used one single latent factor to represent cognitive ability, with six items (the three numeracy items and the three CRT items) as indicators (*λ* ≥ .58), after concluding that using separate factors for numeracy and CRT would have been superfluous; the standardized covariance between the factors was .93 and the superiority in fit of the two-factor model, χ^2^(8) = 13.74, *p* = .089, Δχ^2^(1) = 6.87, *p* = .009, CFI = .996, RMSEA = .027[.000, .050], AIC = 51.74, over the one-factor model, χ^2^(7) = 20.61, *p* = .015, CFI = .992, RMSEA = .036[.015, .056], AIC = 56.61, was negligible. We also used a latent factor to represent religious orientation, with the four items as indicators (*λ* ≥ .49).

We included both bullshit receptivity and profoundness receptivity as predictors and both donation experience and volunteering decision as outcomes. We included cognitive ability, time spent completing the survey, age, education, religiosity, and left-right self-placement as control variables (we excluded sex because it was completely unrelated both to the predictors and to the outcomes), estimating covariances between all predictors and control variables (or error terms in case the case of manifest variables). We gauged the robustness of the results by investigating the effect of removing multivariate outliers on the basis of their Mahalanobis distance from the centroid (*p* < .001; [43]), which yielded virtually identical results. We therefore report results without these exclusions.

When no control variables were included, the model had acceptable fit, χ^2^(100) = 527.5, *p* < .001, CFI = .931, RMSEA = .065[.060, .070]. Consistent with the results of the logistic regression analyses, bullshit receptivity predicted *lower* donation experience, *β* = -.11[-.17, -.04] and volunteering decision, *β* = -.28[-.34, -.21], while profoundness receptivity predicted *higher* donation experience, *β* = .25[.17, .31] and volunteering decision, *β* = .31[.24, .37] (*p* ≤ .001). When all control variables had been added, the fit remained acceptable, χ^2^(367) = 1333.5, *p* < .001, RMSEA = .051[.048, .054] (RMSEA_independence model_ = .154). The path from bullshit receptivity to donation experience was marginally significant, *β* = -.08[-.16, .00] (*p* = .060), and the paths from bullshit-receptivity to volunteering decision, *β* = -.20[-.29, -.12], and from profoundness-receptivity to donation experience, *β* = .14[.05, .22], and volunteering decision, *β* = .22[.14, .30] remained clearly significant (*p* ≤ .002). This model, with all controls included, is illustrated in Figure S1 below.


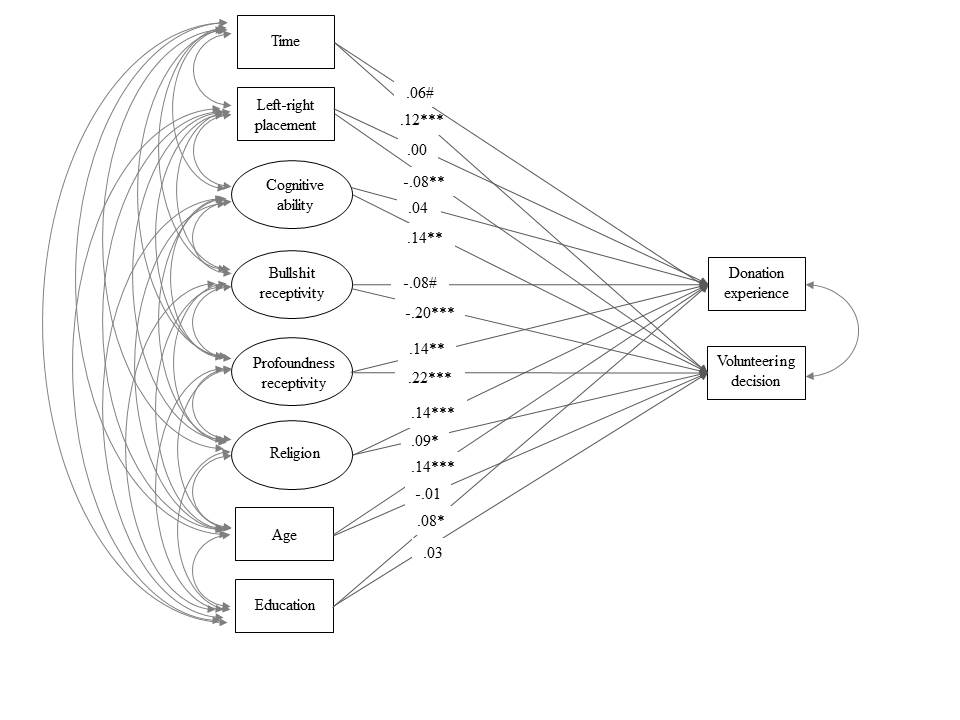


**Fig S1. Structural equation model and standardized parameter estimates from predictors and control variables to donation experience and volunteering decision.** *** *p* < .001, ** *p* < .01, * *p* < .05, # *p* < .10.

# **References for Appendix A**

1. Westfall J, Yarkoni T. Statistically controlling for confounding constructs is harder than you think. PloS One. 2016; 11: doi:10.1371/journal.pone.0152719.
2. Hu LT, Bentler PM. Cutoff criteria for fit indexes in covariance structure analysis: Conventional criteria versus new alternatives. Structural equation modeling: A multidisciplinary journal. 1999; 6: 1-55.
3. Kenny DA. Measuring model fit. Retrieved from http://davidakenny.net/cm/fit.htm. 2015, November 24.
4. Tabachnick BG, Fidell, L. S. Using Multivariate Statistics. 5th ed. Boston: Allyn and Bacon; 2007.
